# Predicting individual differences in reading, spelling and maths in a sample of typically developing children: A study in the perspective of comorbidity

**DOI:** 10.1371/journal.pone.0231937

**Published:** 2020-04-30

**Authors:** Pierluigi Zoccolotti, Maria De Luca, Chiara Valeria Marinelli, Donatella Spinelli

**Affiliations:** 1 Department of Psychology, Sapienza University of Rome, Rome, Italy; 2 Neuropsychology Unit, IRCCS Fondazione Santa Lucia, Rome, Italy; 3 Laboratory of Applied Psychology and Intervention, Department of History, Society and Human Studies, University of Salento, Lecce, Italy; 4 Department of Movement, Human and Health Sciences, Foro Italico University of Rome, Rome, Italy; Universiteit van Amsterdam, NETHERLANDS

## Abstract

We examined reading, spelling, and mathematical skills in an unselected group of 129 Italian fifth graders by testing various cognitive predictors for each behaviour. As dependent variables, we measured performance in behaviours with a clear functional value in everyday life, such as reading a text, spelling under dictation and doing mental and written computations. As predictors, we selected cognitive dimensions having an explicit relation with the target behaviour (called proximal predictors), and prepared various tests in order to select which task had the best predictive power on each behaviour. The aim was to develop a model of proximal predictors of reading (speed and accuracy), spelling (accuracy) and maths (speed and accuracy) characterized by efficacy also in comparison to the prediction based on general cognitive factors (i.e., short-term memory, phonemic verbal fluency, visual perceptual speed, and non-verbal intelligence) and parsimony, pinpointing the role of both common and unique predictors as envisaged in the general perspective of co-morbidity. With one exception (reading accuracy), the proximal predictors models (based on communality analyses) explained a sizeable amount of variance, ranging from 27.5% in the case of calculation (accuracy) to 48.7% of reading (fluency). Models based on general cognitive factors also accounted for some variance (ranging from 6.5% in the case of spelling to 19.5% in the case of reading fluency) but this was appreciably less than that explained by models based on the hypothesized proximal predictors. In general, results confirmed the efficacy of proximal models in predicting reading, spelling and maths although they offered only limited support for common predictors across different learning skills; namely, performance in the Orthographic Decision test entered as a predictor of both reading and spelling indicating that a single orthographic lexicon may account for performance in reading and spelling. Possible lines of research to expand on this approach are illustrated.

## Introduction

Problems in learning to read, spell or perform maths tasks are referred to as “specific” because they occur in the absence of deficits in general cognitive skills (intelligence) and neurological disorders, and in spite of regular involvement in school activities. A great deal of research has been carried out on the cognitive antecedents of specific learning disabilities (SLD) encompassing different approaches and perspectives. This research has, however, typically focused on only one of these developmental disorders (whether reading, spelling or maths). A key problem with this approach is that it neglects the presence of widespread co-morbidities among learning disorders [1] as well as with other developmental disorders, such as oral language deficits or attention-deficit/hyperactive disorder (ADHD) [2].

Recently, research on developmental co-morbidities has made important advances and clearly indicates that co-morbidity is a common phenomenon; thus, co-occurrence of disorders does not only concern “close” disorders within a given domain (or homotypic co-morbidity) but also occurs among “distant” disorders (or heterotypic co-morbidity) [3]. In a large series of studies, Pennington and co-workers (e.g., [4,5]) examined the homotypic comorbidity between the reading disorders and the speech-sound disorder, and the heterotypic comorbidity between the reading disorders and ADHD. Indeed, in both cases, there was substantial overlap among disorders which undermines the idea that closeness of cognitive domains may be crucial in understanding these associations (for an early synthesis of these studies see [2]).

Traditional cognitive research is not well suited to deal with co-morbidities because it pursued a narrow perspective focused on single deficits. A clear example of this is the development of cognitive architectures in reading. These started from different theoretical perspectives. On one hand, the well-known dual route model of reading, which is based on a separation between lexical and non-lexical routes, was developed and updated (e.g., [6]; named dual route cascaded model or DRC by [7]) and has influenced more recent models (such as, for example, the CDP+ by [8]). On the other hand, other models, such as the triangle model [9,10], were developed in a connectionist perspective and refer to a single processing procedure. However, it must be observed that none of these models can easily accommodate the presence of co-morbidities between reading and other developmental deficits because they focus on the specificity of the reading process; they try to make explicit the cognitive processes that are selectively active in order to account for different aspects of reading.

In the present study, we aimed to develop and test a cognitive architecture that would consider reading, spelling and doing maths unitarily. As described in detail below we carried out an initial test of this model on a sample of typically developing children. The long-term interest is that of developing a model that would be able to account for the co-morbidities among SLD. Below we present the arguments for positing a proximal model of learning. Note that the relevant literature is largely based on studies on children with SLD; so, even though our study is on typically developing children we will draw heavily on studies on children with atypical development.

### “Proximal” and “distal” approaches

The distinction between proximal and distal causes is central to the development of cognitive architectures (e.g., [11]). Proximal processes are described as the cognitive antecedents of reading and in particular as “*causes of poor reading performance that consists of some abnormality of the child’s cognitive reading system*” [11]. In this perspective, a model of reading (spelling or maths) is a complex architecture featuring the relationships between these proximal antecedents and reading. A wealth of more general cognitive processes (such as short- and long-term memory, perception, attention etc.) are evidently necessary to support the activity of the processes envisaged by the model but they are seen as distal processes to the extent to which no explicit relationship is put forward with the reading behaviour and with the proximal factors considered in the architecture of reading. In terms, their role on the target behaviour is expected to act indirectly to the extent in which they influence some of the proximal processes envisaged within the cognitive architecture of reading [11].

Besides the narrower perspective focused on proximal processes, a long-lasting tradition of studies, aimed to account for reading (but also spelling and maths) deficits from a broader cognitive perspective, examined whether children with dyslexia have deficits in short-term memory (e.g., [12]), executive processing (e.g., [13]), attention (e.g., [14]), rapid naming (e.g., [15]), phonological awareness (e.g., [16]), etc. These investigations can be broadly framed as studies examining the distal cognitive antecedents of SLD [11]. One strength of this approach is that it is not limited by the very specific constraints of cognitive architectures; thus, it is potentially open to examining relationships between seemingly distant phenomena (as it is the case for developmental heterotypic co-morbidities). One weakness is that broad categories, such as attention or memory, can influence complex behaviours, such as reading, spelling or doing maths, in different ways but these relationships remain implicit in a distal perspective.

Thus, it is quite difficult to provide a theoretical synthesis of the studies examining the distal cognitive antecedents of SLD. Indeed, if one looks at the panorama of cognitive theories of dyslexia (i.e., it is a temporal processing deficit, a procedural deficit, a phonological deficit, a perceptual deficit, etc.), one is surprised by the variety of possible interpretations, impinging on a large variety of cognitive domains (such as time perception, executive functions, etc.).

A similar, though not identical, distinction that can be used to frame studies on cognitive antecedents of learning skills is that between domain-specific vs. domain-general processes. Indeed, distal processes tend to refer to broad, domain-general, categories (such as memory, attention etc.) while proximal processes generally indicate domain-specific processes (e.g. [17]). However, the proximal-distal dichotomy refers more explicitly to a formal architecture of the reading (spelling, maths) not only to the breadth of the processes considered.

Overall, on the one hand, in a distal perspective, causes of reading (spelling or maths) disorders are put forward without explicit reference to cognitive architectures of reading (spelling or maths); on the other hand, the proximal approach featuring direct relationships with reading processes seems more effective but excludes *a priori* the possibility of explaining co-morbidities between reading and other developmental deficits.

### Proximal approach to the study of comorbidity

Can the proximal approach be reformulated and extended in order to tackle co-morbid disorders? As stated above, the traditional cognitivist way to model learning disorders has been largely focused on single deficits. Pennington [2] expressed his concern over this approach by stating that “*At the same time that a probabilistic*, *multifactorial model of the etiologies of these disorders is widely accepted*, *our cognitive analyses of them often relies on a deterministic*, *single deficit model*. *So*, *there is a potential contradiction between our etiological and cognitive models for understanding such disorders*” (p. 386).

Notably, there are a few instances in which the question of how to account for a plurality of deficits has been examined in the cognitive literature. One example is the controversy as to whether separate orthographic lexica have to be considered to account for reading and spelling, or whether one can imagine that reading and spelling both impinge on the same lexicon and that, as a consequence, a deficit at the lexical level will affect both reading and spelling (for a discussion see [18]). The question is still open but here we would like to stress that the idea of referring to a single lexicon for reading and spelling represents an interesting reference within the perspective advanced by Pennington [2]. In particular, the multiple deficit model proposed by this author states that “*comorbidity among complex behavioural disorders is to be expected because of shared etiologic and cognitive risk factors*” (p. 404). Thus, assuming the presence of a single lexicon for reading and spelling might account at least in part for the presence of the (frequent) association between reading and spelling deficits.

At the same time, the presence of two partially different architectures also provides the ground for understanding the presence of dissociations between reading and spelling deficits, as reported in both acquired (e.g., [19]) and developmental cases (e.g., [20]). This example indicates that in principle it is possible to tackle developmental co-morbid deficits by means of cognitive architectures in a proximal perspective. Yet, it must also be noted that dyslexia and dysgraphia are very “close” (or homotypic) deficits and it is still to be proven that this approach can be extended to more “distant” (or heterotypic) co-morbidities (such as reading and maths).

### Selecting the target behaviour: Reading single word vs. functional reading

An additional feature, which could undermine the possibility of cognitive models to account for co-morbid deficits (defined by Pennington as associations among “complex behaviours” [2]), is that they typically focus on a very simplified, abstract, non-ecological condition of the target behaviour. For instance, models (such as the DRC model) aim to predict performance in single word reading aloud in English. There is good reason for choosing the word level as a target for modelling reading. Word recognition is impaired in dyslexia (both acquired or developmental) whereas simpler stimuli (such as letters) are usually (though not always) processed well [21,22,23,24].

However, fluent reading in a functional context, such as reading a text passage, requires more than efficient single word processing. Indeed, to read a text, the reader has to synchronize a number of ongoing processes such as visual scanning, recognition, short-term memory, and pronunciation [25,26,27]. Thus, even in the absence of specific speed instructions, reading a text is a task that inherently poses tight time constraints and requires synchronization of processing to the observers. There is evidence that typically developing children acquire the ability to process multiple written stimuli (i.e., arrays of words such as passages) presumably between first and third grade (i.e., after recognition at the single word level is well established [28,29]) and that children with dyslexia are specifically impaired in processing orthographic stimuli presented in multiple displays as compared to single displays [30]. The deficit in processing targets from multiple displays is still poorly understood. Yet, it seems well established that children with dyslexia do not show deficits in the sub-components involved in processing per se. For example, they are not impaired in programming saccades [31] or in making corrective movements after sub-optimal landing positions [32].

Overall, using reading a meaningful text as target behaviour appears a more ecological, less abstract condition with respect to single word reading. Furthermore, it should be kept in mind that co-morbidity is expected among “complex” behaviours [2] and an analysis based on a less natural task (such as single word reading) might fail to capture this phenomenon.

### Functional reading as target behaviour: RAN as a proximal factor

The ability to process multiple targets is an important part of the reading process and might contribute to the dyslexic deficit over and above the deficit at the word level; nevertheless, it has been neglected for a long time in favour of studies focused on single words. One task that might capture multiple targets processing ability is the well-known Rapid Automatized Naming or RAN [33,34]. In this task, the child has to quickly scan and name a large set of familiar targets (typically colours, objects, or numbers). It is well established that the performance of children with dyslexia is impaired in RAN tasks, although there are several different interpretations of this relationship (for reviews see [15,35,36]). So, some authors have proposed that RAN is just another example of a phonological task (e.g., [37]) or that it points to deficits in lexical access or retrieval (e.g., [38]). A crucial finding in the literature is that the typical slowness of children with dyslexia with respect to typically developing readers is selective for serial RAN. If stimuli (colours, objects, or numbers) are singly presented, children with dyslexia are not, or only minimally, impaired, and performance on discrete (single-item) RAN trials does not correlate with reading fluency (e.g., [39]).

Thus, we have proposed that RAN tasks mark the ability “*to integrate the sub-components involved in reading from multiple displays*”, which is typical of successful reading [40]. This hypothesis makes it explicit the earlier idea put forward by Wolf and Bowers that RAN is “*a microcosm of reading*” to the extent to which it requires all the processes involved in fluent reading, except for orthographic decoding [35,36]. One important feature of this proposal is that, unlike what has been done up until now, it interprets RAN in “proximal” terms, i.e., it makes it explicit the contribution of processes marked by RAN to reading, and reading from multiple word displays in particular, as typical of functional reading in everyday conditions. Evidence in support of the present proximal interpretation of RAN comes from studies indicating that RAN makes an independent contribution to the prediction of reading fluency over and above measures of orthographic processing [29,40].

It seems important to observe that tasks (such as RAN) are neither proximal nor distal per se. It is the relationship envisaged with the target behaviour that may be expressed in proximal or distal terms. Therefore, considering a task in proximal or distal terms depends on the theoretical perspective of a given study. Here we will consider this possibility and make hypotheses about the potential proximal nature of predictors even though in the literature they have not always been considered in this way, i.e., they were used without making explicit the relationship between the predictor and the dependent variable.

### Associations and dissociations of reading and spelling

Proposing that reading is a task with high time constraints due to the need to synchronize a number of ongoing processes provides a framework for interpreting possible dissociations between reading and spelling. Indeed, spelling, which requires activation of orthographic and hand-motor processes, poses lesser time constraints and synchronization with respect to reading, and one does not expect that processes representing repetitive speeded processing, such as RAN, have a role in this case. On the contrary, spelling may also place particular constraints on the quality of the phonological representations to be converted into written output both dictation and spontaneous writing. Indeed, several authors posited that efficient spelling requires fully specified phonological representations while reading may also take advantage of partial cue processing [41,42]. This brief analysis creates the premises for an interpretation of reading and spelling associations and dissociations as actually observed in clinical practice in terms of shared and independent processes. Indeed, one working hypothesis could be that an efficient lexicon would be instrumental to both reading and spelling, whereas performance on RAN could contribute to predictions about reading but not spelling. Some evidence goes in this direction (e.g., [43]). Conversely, efficient phonological processing might contribute predominantly (or only) to spelling and less (or not) to reading, particularly in the case of reading speed [44].

In this vein, note that it is controversial whether phonological or meta-phonological processes should be viewed in proximal or distal terms. In his analysis of this question, Coltheart [11] makes an in-depth analysis of the role of phonological awareness in reading and concludes that the identification of the proximal/distal nature of a variable depends on the theoretical interpretation of its relationship to the target behaviour. In one (distal) perspective, phonological awareness is seen as important for learning to read but not for identifying processes used directly in the online analysis of words while reading. By contrast, if one sees (however hypothetically) the role of a given phonological (or meta-phonological) process as directly involved in the online analysis of a given stimulus, this would mark the variable in proximal terms. Therefore, following Coltheart’s analysis [11], one should make this theoretical formulation case by case by analysing the processing required in a given stimulus/task condition. In this perspective, we propose that, in the case of spelling, the ability to form and hold in memory an efficient phonological representation (as assessed by appropriate task conditions, such as non-word repetition and phonemic segmentation) should be seen as a proximal predictor. In particular, it appears that in order to identify the correct orthographic pattern and spell a word the child makes continuous reference to its phonological representation, which is actively maintained in working memory.

### Comorbidity of reading and maths deficits

A few studies considered the comorbidity of reading and maths deficits. In children, Slot, van Viersen, de Bree, and Kroesbergen [45] observed that phonological awareness, alphanumeric and non-alphanumeric RAN and verbal short-term memory predicted literacy; by contrast, number sense, visual-spatial working memory and phonological awareness predicted maths. Thus, phonological processing was shared in both behaviours, explaining comorbidity. In adults, it has been reported that individuals with dyslexia and dyscalculia share domain general deficits in rapid naming and verbal short-term memory, but it is unclear whether these associations are sufficient to explain their co-morbidity [46]. Recently, it was reported that deficits in visual perception (and in particular in numerosity processing) were common in children with dyslexia and dyscalculia [47].

These studies provide relevant information to tackle the comorbidity problem and we will take into account their results; however, a weakness, characteristic of the distal approach, is that they generally fail to specify the relationship between predictors and target behaviours with reference to the corresponding cognitive architectures.

Overall, we propose the potential heuristic value to envisage an analysis of developmental co-morbidities based on cognitive models that, in a proximal perspective, specify processes which exert independent and shared influences on target behaviours with reference to a unitary architecture of reading, spelling and maths. Below, we will outline such an architecture; then, we will present a study carried out in a group of typically developing children aimed to provide an initial test of this theoretical framework.

### Towards a proximal model of reading, spelling and maths

**Fig 1:**
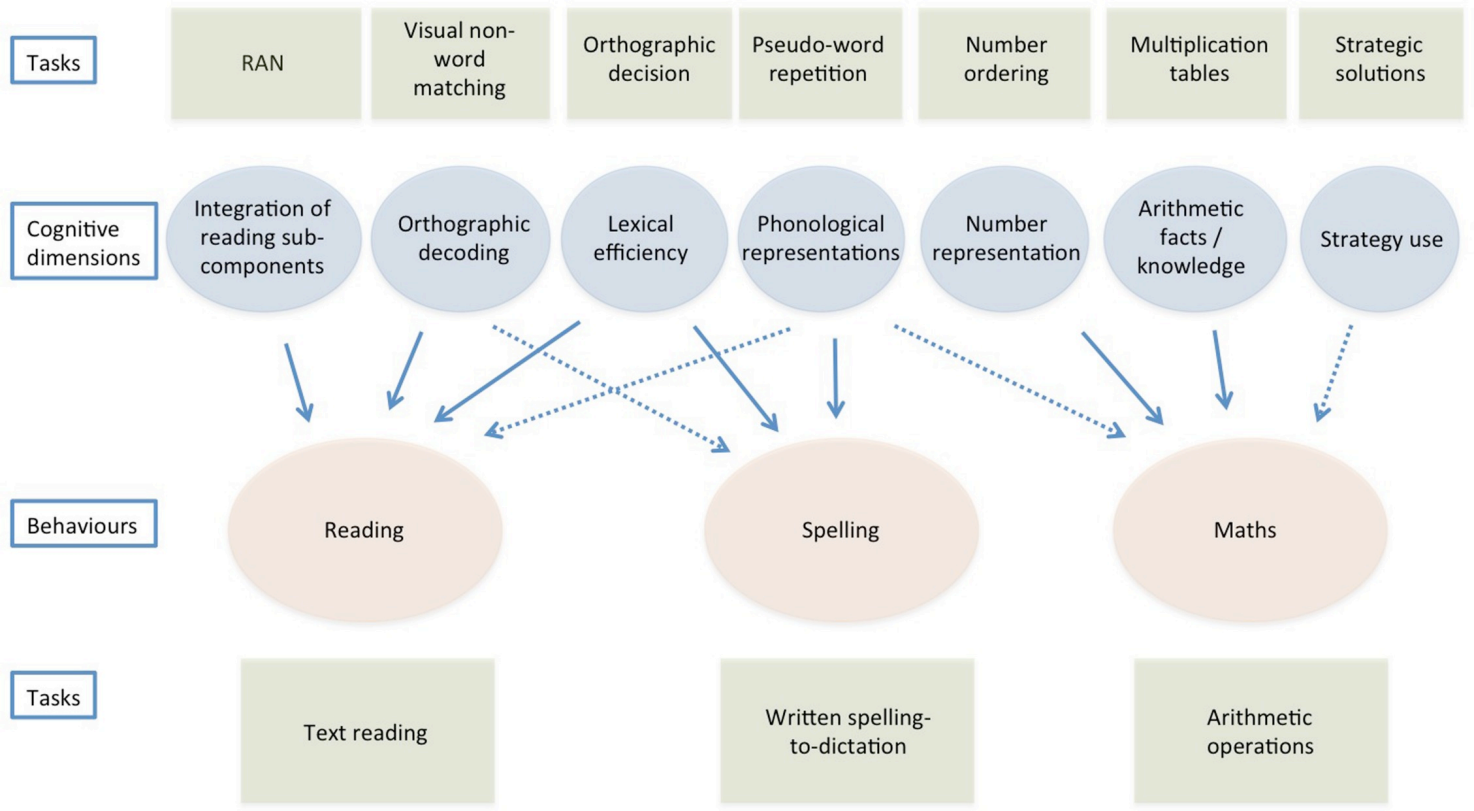
A schematic proximal model of reading, spelling, and doing maths. The model illustrates the hypothesized cognitive dimensions and their putative links to reading, spelling, and doing maths. Note that links may be unique or shared with different behaviours. Continuous lines indicate expected associations; dotted lines indicate less certain relationships. Some examples of the tasks referring to cognitive dimension (top) and behaviours (bottom) are presented in the figure.

Fig 1 illustrates a reference schema of the key cognitive dimensions (in light blue) that may influence the behaviours (in light red) regarding reading, spelling and doing maths (in light red), and some possible unique or shared links (indicated by the arrows). The schema also specifies (in light green) some of the tasks used to measure each hypothesised cognitive dimension (top), as well as tasks used to measure each target behaviour (bottom).

Following is the full list of actual tests used to measure predictors: RAN colours and RAN digits (Integration of reading sub-components); Visual-visual, Visual-auditory, and Auditory-auditory non-word matching (Orthographic decoding); Orthographic decision task (Lexical efficiency); Single Pseudo-word Repetition, Single Pseudo-word Phonemic Segmentation, Repetition of Pseudo-word Series (Phonological representations); Number order (Number representation), Arithmetic facts (Arithmetic facts/knowledge), Computation Strategies (Strategy use), Dictation of numbers, Arabic number reading, Computation procedures (Control arithmetic tasks).

The considerations specified above suggest selecting as target behaviours tasks with a clear functional value in everyday life, close to those experienced by children at school. Note that this perspective is different from the traditional cognitive modelling in which the target behaviour is a non-ecological, abstract task, such as single word reading aloud. Accordingly, we do not refer directly to such models in framing research with the perspective of predicting co-morbidities of learning disabilities.

With regard to reading and spelling, the predicted relationships between cognitive dimensions and behaviours are based on the available evidence concerning regular orthographies, particularly in Italian—the object of the present study. For maths, hypotheses are based on the current scientific literature, which we tried to frame in proximal terms, i.e., by selecting specific cognitive dimensions, which might, at least on logical grounds, make independent contributions to an architecture of maths skills. In Fig 1, we used continuous lines to indicate associations for which there seems to be convincing supportive evidence and with dotted lines to indicate relationships for which evidence is mixed. Note that one important goal of building a cognitive architecture model for comorbidity is to keep the number of factors low, considering only factors that, at least on logical grounds, should provide a mostly independent contribution. Further consider that, at this point of analysis, our proposal of proximal predictors is largely based on hypothetical considerations and that the process is highly heuristic in its nature.

As for reading, based on previous evidence [40], we expected that orthographic decoding and integration of reading sub-components would make independent contributions to the prediction of reading a meaningful passage. While in that study we considered only these two factors there is also convincing evidence [48,49,50,51,52] that lexical efficiency is an important factor even in regular orthographies, such as Italian (for similar considerations on German see [53]). In fact, data indicate that, by third grade, typically developing children show sensitivity to word frequency [54,55]; furthermore, lexical effects have been shown even in the reading of pseudo-words [56]. Therefore, at least three logically independent factors might contribute in predicting reading fluency, and some interaction between orthographic decoding and lexical efficiency might also be expected. Quality of phonological representations is a further option; however, previous evidence on Italian on this is mixed. In particular, it has been found [57,58,59] that only children with dyslexia who have a previous history of language delay are impaired in phonological and meta-phonological tasks, making the prediction more uncertain (in the figure this uncertainty is shown with the use of a dotted line). Similarly, it has been reported in German-speaking children that deficits in phoneme processing are not central in dyslexia. More recent evidence indicates a role of phonological processing for reading accuracy, but not speed, across five different languages [44].

Note that predictions for Italian children are spelled out for reading fluency (see Fig 1) while they may not necessarily apply to reading accuracy. Indeed, it has been claimed that, in regular orthographies, fluency is a much more sensitive measure of reading performance [60,61]. In part, this may depend upon the presence of ceiling effects in accuracy, which is already quite high by the end of first grade in the case of regular orthographies [62]. At any rate, it seems unlikely that RAN tasks would be relevant in predicting reading accuracy; thus, prediction of reading accuracy will exclude the factor integration of reading sub-components (measured by RAN).

In the case of spelling, there is evidence in support of the idea that lexical efficiency plays an important role [48,63,64]. Furthermore, as described above, we expect that the quality of phonological representations and processing makes an important contribution to the prediction of spelling skills [44,65], especially in children with previous language deficit [66]. Unlike reading, there is no clear evidence that orthographic decoding is critical in the case of spelling [67]. Thus, Fig 1 reports lexical efficiency and phonological representation as cognitive predictors of spelling while the link between orthographic decoding and spelling is considered as more uncertain (and henceforth marked with a dotted line).

In the case of maths, the current literature provides a set of alternative interpretations of how the process works. One dominant line of research emphasises the ability to represent and process numerosity as the key factor in accounting for numerical skills [68,69,70]. The development of mathematical skills can be seen as “*an increasingly sophisticated understanding of numerosity and its implication*, *and an increasing skill in manipulating numerosity*” (p. 3, [68]). This ability is likely based on a “*number sense*” considered as an approximate representation of numerosity in animals and preverbal humans [71]. The acquisition of numeric symbols involves this core system for numerosity (generally tested with the presentation of dot displays) but requires a refinement, possibly connected with linguistic representations for numbers [72]. Many studies focused on the association between approximate numerosity acuity and mathematical learning achievement/disability [73,74,75,76,77,78,79]. However, much negative evidence was also reported [77,80,81,82,83]. Contrasting results may be due to differences across development to the extent to which symbolic (digits) and non-symbolic (dots) tasks reflect the precision of the approximate numerosity system or, alternatively, to the existence of two distinct representational systems (e.g., [83]). In any case, the relationship between symbolic comparison task performance and maths achievement is robust [84]. As our interest is in making explicit logically independent proximal predictors of mathematic processing, here we limit our formulation of number representation to symbolic stimuli. Also, it has been reported that adults dyslexic have a deficit in maths tests requiring symbolic comparison, not in non-verbal magnitude representation [85].

In addition to the putative role of number representation, much evidence also points to an independent contribution of knowledge of arithmetical facts (for a review see [86]). Dyscalculic children have difficulties in learning and remembering arithmetic facts, and in executing calculation procedures, with long solution times and high error rates [87]. Geary and colleagues [88,89] suggested that semantic memory difficulties may explain these learning difficulties. However, there is little empirical evidence for a non-numerical semantic deficit in dyscalculic children [90]. Thus, a specific learning difficulty seems to be present, possibly due to a working memory deficit [91]. With sufficient practice, children learn specific responses to frequently occurring operations: a typical example is multiplication. In this way, they pass from the use of an algorithm (slow procedure, such as counting) to the direct access to a numerical solution (fast procedure, based on memory retrieval). According to many authors (e.g., [87]), this knowledge of arithmetic facts is reduced in dyscalculic children and is included in the diagnostic criteria of international manuals [92]. There is reason to believe that performance on tasks that require the retrieval of arithmetic facts may be partially independent from performance on tasks that require an evaluation of numerical magnitudes (e.g., [38]). Thus, in the schematic model in Fig 1, we consider both a dimension of “number representation” and a separate dimension of “arithmetic fact knowledge”.

Finally, it has been emphasised that the acquisition of numerical skills also passes through the development of active strategies in solving arithmetic tasks [93]. Strategies may entail the appropriate choice between relying on arithmetic facts and using an algorithm; they may also express themselves in the flexible use of these two procedures, so that relatively complex problems are fractionated into smaller problems so as to use an effective mixture of arithmetic fact retrieval and algorithms [86]. For instance, if we look at De Smedt’s [86] example, the sum 6 + 8, can be decomposed into two separate problems that the subject can easily solve by retrieval: 6 + 4 = 10, and 10 + 4 = 14. This ability to decompose a number in order to facilitate the task is “strategic” in the sense that it optimizes the processing resources of the subject. However, note that this strategy would not be possible if there were no direct retrieval of arithmetic facts (such as 8 as the sum of 4 + 4). In this case, strategy would indicate the ability to see 8 as a sum of two convenient-to-manipulate quantities in relation to the general goal (add to 6; add to 10). Although retrieval of arithmetic facts, strategy and number representation are strictly intertwined, it seems possible that using strategies, such as fractionating a problem, might be seen as at least partially independent from number representation and arithmetic fact knowledge. Therefore, in the schematic model in Fig 1, we tentatively included a third variable, i.e., “strategy use”, marking this link with a dotted line to indicate its provisional nature. Other factors may contribute to mathematical performance but these three factors seem the most promising to account for a sizeable number of calculation skills; and for this reason number representation, knowledge of arithmetic facts and strategy use are depicted in Fig 1 as independent cognitive predictors.

In any case, the presence of alternative interpretations for dyscalculia should be acknowledged (for a discussion see [94]). For example, Szucs and co-workers [95] claimed that short-term visual-spatial memory and inhibition might be critical for distinguishing between children with and without numerical deficits, after controlling for several factors including age and IQ. While this evidence seems quite strong, it is difficult to frame it within the proximal approach developed here (we will check some of these distal hypotheses testing a “control” model; see at the end of section “Study design: Control tests”). Working memory difficulties were also proposed [87]. However, results were conflicting and Landerl and co-workers [69] concluded that, even if working memory difficulties are present in dyscalculia, no convincing evidence indicates that working memory is a causal factor of the disorder. In the present study, we will also check for this distal hypothesis. Other studies have reported associations between phonological awareness and mathematical achievement or mathematical difficulty [45,46,96,97,98,99,100,101,102]. According to some authors, phonological awareness could also be a cognitive factor that accounts for the co-morbidity between mathematical deficits and reading/spelling deficits, although this evidence is still scanty [17,45,46,103,104].

Since the present study aims to develop a proximal model of comorbidity, it seems that the quality of phonological representations can represent the factor active in all three behaviours and we will check this dimension also for maths. It has been proposed that phonological ability might have a role in maths performance because it is involved in translating an Arabic number into a verbal code [98,105,106], counting [107], or retrieving a phonologically based code from long-term memory when answering a question (such as 3 x 2 = 6). However, as the association between phonological ability and maths seems less strict than in the case of spelling, this association is marked with a dotted line in Fig 1, similarly to the association proposed with a dotted line in the case of reading.

Overall, the scheme presented in Fig 1 is a handy reference for framing research on the possible role of cognitive dimensions in accounting for the partial associations existing among reading, spelling and maths skills in real life conditions but does not claim to cover all processes involved in these complex behaviours.

### Study design: Identifying dependent and independent variables for testing proximal models of reading, spelling and maths

We examined reading, spelling and maths skills in an unselected sample of Italian fifth graders. As it is well known, performance in reading, spelling and calculation is described by continuous distributions and so-called atypical performances merely indicate those lying at the very low ends of these continuous distributions [2,108]. Thus, one should expect that associations and dissociations among key behaviours can be demonstrated in both “normal” and “extreme” ranges of performance.

As dependent variables, we used measures that have a clear functional value in everyday life. Therefore, in the case of reading, we measured the time required to read a meaningful text aloud, such as a text passage. Fluency on such stimulus materials is relevant in the everyday life of the child. In this context, we also evaluated reading accuracy; however, this parameter is somewhat less sensitive to reading performance in a highly regular orthography, such as Italian (see section on Reading accuracy). As for spelling, we considered spelling accuracy. Children were asked to spell from dictation a meaningful passage that included words with different levels of complexity and regularity. Finally, concerning maths, we measured performance on a variety of calculation tasks (including all four operations); measures of both accuracy and speed were considered.

Independent variables, i.e., cognitive dimensions, were broadly modelled on the scheme described in Fig 1. The definition of “cognitive predictors”, represented by the link between cognitive dimensions and behaviours in the figure, derived from hypotheses that were characterised by different degrees of specificity both in terms of the dimensions (supported by the considerations on the literature described in the above sections) and of the markers derived from the tasks. For example, for reading, our hypotheses predict that three different cognitive dimensions (also referred to as factors) have a role: orthographic decoding, integration of reading sub-components, and lexical efficiency. The tasks, and consequently the markers selected in the study to measure these factors were multiple; and the ability of the tasks/markers to predict the behaviours was also under investigation. For example, in the case of orthographic decoding, we developed new tasks for measuring orthographic decoding based on a two-alternative choice that involved the same-different comparison between two pseudo-words. Since there is some evidence that the key decoding process may concern an orthographic–phonological binding [109], stimuli could appear in a visual-visual mode of presentation as well as in a visual-auditory mode (an auditory-auditory modality was also used as a control).

The same general approach was used to develop tasks for all target behaviours considered. As for spelling, we expected a specific influence of the lexical efficiency and quality of phonological representations dimensions (or factors) and a lesser role for orthographic decoding. Also, to test the quality of the dimension of phonological representations we used different tests, including different forms of the non-word repetition task and a phonemic segmentation task. As for maths, we expected a specific influence of the number representation, knowledge of arithmetic facts and strategy use dimensions (or factors); we also added the role of the quality of the phonological representations that was somewhat more uncertain. To test these dimensions, we used a number of specifically designed tasks (see caption of Fig 1); moreover, we included a number of control tasks, including knowledge of computation procedures and number reading.

### Study design: Control tests

Overall, the aim of the model was to uncover unique (and shared) influences of predictors for which it is possible to frame a proximal relationship between predictors and target dependent measures. However, as a control we thought it might also be interesting to examine the putative role of general cognitive factors, which have been associated in varying degrees to reading, spelling and calculation. In particular, we considered measures of short-term memory, phonemic verbal fluency, visual perceptual speed and non-verbal intelligence. Such measures have been variously associated with performance in reading, spelling and maths tasks. For example, it has been reported that performance in short-term memory is predictive of both literacy and maths skills [109,110]. Verbal fluency has been found impaired particularly in children with disphonetic dyslexia (e.g., [111]). Visual perceptual speed has been found to be impaired in children with dyslexia in a variety of studies by Pennington and colleagues (e.g., [4]). Finally, it has been reported that performance in the Raven Progressive matrices predicts maths and comprehension tests independent of sociocultural level [112]. Within the terminology adopted here, these are “distal” predictors in the sense that some relationships with the dependent measures are expected; however the nature of these relationships is not specified in terms of the cognitive architectures of the target behaviours and they may occur through complex interrelationships with the cognitive proximal predictors. We tested the possible predictive power of general cognitive dimensions in two different ways. Firstly, we used these general dimensions to build a model of the target behaviour; comparing this model to that stemming from an analysis of the proximal predictors should provide information on the specificity and efficiency of proximal models. Second, we added the general cognitive dimensions to the various proximal models (of reading, spelling and maths) to test whether these variables allowed increasing the general power of the original model.

## Materials and methods

### Participants

An unselected sample of 129 (65 male, 64 female) Italian children (mean age = 10.7 years; SD = 0.3; range = 10.1–11.3 years) were examined. All children were fifth-graders, they performed adequately on Raven's CPM [113] and had normal socio-educational conditions. All participants were natives of the areas in or around Rome and were tested at their own schools.

Parents were informed about the screening activities and authorised their child’s participation by signing the appropriate informed consent paperwork. The study was carried out in accordance with the principles of the Declaration of Helsinki and was approved by the ethical committee of the IRCSS Fondazione Santa Lucia and by the school authorities.

### Materials

Below we describe the materials by grouping the test(s) that measure the dependent variable(s) and the specific predictors for each of the three target behaviours. In the case of the novel tests devised for the present study, information on stimuli and task characteristics is described in greater detail in S1 Appendix.

#### Reading measures

The dependent variables for reading were based on the *MT reading test* [114]. In this standard test, the child reads a text passage aloud with a 4-min time limit. Reading time in seconds per syllable is measured. Reading accuracy is evaluated based on the number of errors. Scoring takes into account the functional meaning of errors. Each word with an elision, substitution, insertion or inversion of letters is scored as 1 error, while changes in stress assignment, spontaneous self-corrections, errors that do not change the meaning of the text, repetitions of the same errors and hesitations are given a 1/2 score. If the child does not complete the passage reading, the number of errors adjusted for the amount of text read is scored. Both speed and accuracy measures were used in separate communality analyses.

Performances in the following tests were considered as predictors of the reading performance.

*RAN* [115,116]. The test consists of matrices of colors (test 2) or digits (test 3), printed on sheets of paper. Two different matrices are generated for each condition. Five different elements are presented for each of the two stimulus conditions. The colors are small 1 x 1 cm squares; they are black, blue (RGB 51–102–255), red (RGB 221–8–6), yellow (RGB 252–243–5) and green (RGB 31–183–20). The digits (Helvetica font, size 24) are the numbers 2, 4, 6, 7, and 9. In each matrix, there are 10 rows of five stimuli for a total of 50; the sequence of the stimuli is randomized within each matrix with the constraint that no more than two identical items could be presented consecutively. A practice trial with a 20-stimuli matrix is also arranged to allow familiarization with the test. The order of presentation of the conditions is colors, then digits. The child is shown one matrix at a time, placed on a flat surface approximately 40 cm away from he/she. The participant is requested to name each stimulus on the matrix as quickly and as accurately as possible, working from left to right, by rows. The practice trial is run before each corresponding condition. During this trial, the examiner corrects any error made by the child. Unfamiliar or colloquial expressions are corrected so that all participants use the same terms during the actual test. If necessary, the practice trial is replicated. The time to complete the task is measured separately for each matrix using a stopwatch. The dependent measure is the time in sec. per item, based on the total time over the two matrices (50+50 items), separately for colors and digits. Errors (such as naming errors, omissions, or target inversions) are also measured. However, only time measures (sec. per item) were used for the present analyses.

*Orthographic decoding*. *(visual-visual*, *visual-auditory and auditory-auditory pseudo-word matching tests)*. The child has to say whether or not two pseudo-words presented in the visual modality (test 4), in a mixed visual-auditory modality (test 5) or in the auditory-auditory presentation (test 6) are the same or not. The three experimental tasks were created in our laboratory in order to evaluate the ability of orthographic decoding. The tasks require the child to make a comparison between two pseudo-words in the visual modality (on the basis of their orthography) or in a mixed visual-auditory modality (on the basis of the correspondence between orthographic and phonological information). In a control condition (based on an auditory-auditory presentation) the child has to make a comparison between two pseudo-words on the basis of their phonological structure). In all cases, the child has to indicate whether the two pseudo-words are the same by crossing a YES/NO response box on a paper response sheet. Depending on the task, the distracters are phonologically or orthographically similar (see below for details). The response whether or not pseudo-words are the same cannot be accomplished by pronouncing them because reading aloud is not allowed, or by purely visual comparison (in the visual-visual task) since one item is written in upper-case letters and the other one in lower-case. In the same vein, the response in the auditory-auditory task cannot be based on a purely auditory comparison of idiosyncratic vocal/speech cues as two different persons pronounce the two items of the pair. Accuracy is measured.

*Orthographic decision*. The child has to read silently a list of high- and low-frequency inconsistently spelled words (and corresponding derived pseudo-homophones, homophonic to the original words but orthographically incorrect for the presence of a plausible phonological error) and to indicate whether or not they are correctly spelled. The number of errors in judging both correct words and pseudo-homophones is scored.

#### Spelling measures

The dependent measure for spelling was evaluated by standard test *"Nonna Concetta" Spelling-to-dictation* [117]. The task is a spelling to dictation test, consisting in a meaningful passage that includes words with regular and unpredictable spelling, tapping the efficiency of both lexical and non-lexical spelling procedures. Modality of administration is standardized. The experimenter reads the meaningful passage, following the pauses established by the test; usually, two or three words are read at a time. The child has to spell the text on a white paper. The scoring is made by calculating the total number of elements for which a mistake was present.

Performances on the following tests were considered as predictors of the performance in spelling.

*Single pseudo-word repetition and phonemic segmentation*. A list of long pseudo-words is presented in the auditory modality. The child has to repeat the stimulus and then segment it. The number of correct repetitions and correct segmentations were scored.

*Orthographic decision*. See description above.

*Repetition of pseudo-word series* [118]. This test is comprised of 10 series of triplets of 3- and 5-letter pseudo-words presented in the auditory modality. The pseudo-words were read aloud by a speech therapist and recorded and processed using the Audacity 2.0.2 software. The resulting audio file (.aup format) is used during the test administration using the same software. The structure of the presentation of each triplet of non-words in a trial is as follows: a non-word is delivered every 2 seconds; after the third one, an acoustic warning (beep) occurs (300 ms after the pronunciation offset); the experimenter stops the reproduction of the audio file upon the warning to allow the child repeating the triplet; then the experimenter releases the reproduction of the file to let the child listen to the following triplet, and so on. The child is asked to listen to the triplet and repeat it in the same order immediately after the acoustic warning. The number of correctly repeated non-words (out of 30, independent of the sequence order) was scored.

#### Maths: Numerical skills measures

The dependent variables measuring numerical skills were based on the following two standard tests:

*Mental Calculation (from the AC-MT 6–11 battery* [119]*)*. This is a sub-test from the AC-MT 6–11 years Test for evaluation of calculation abilities [119]. The child performs three sums and three subtractions of two two-digit numbers in the mind as quickly and as accurately as possible. One point score is given for every correct calculation. If the child does not respond within 30 seconds, an error is considered. The time to execute the task is scored from the moment the examiner has finished to pronounce each calculation to be performed until the child gives back the result. The number of errors over 6 trials is scored as well as the time to perform the task (time in seconds per trial).

*Arithmetic calculations (from the AC-MT 6–11 battery* [119]*)*. The child performs two calculations for each of the four basic number operations, based on two numbers. The number length varies from 3 to 5 digits, and some numbers may include decimals. One point score is given for every correct calculation. The number of errors over 8 trials is calculated. This battery considers an accuracy score derived from both the mental and written arithmetic calculations and a time score, derived only by the mental calculation test. These two scores were the dependent measures for the analysis of mathematical skills.

Performances on the following tests were considered as predictors of performance in maths.

*Dictation of numbers (from the AC-MT 6–11 battery* [119]*)*. The child has to spell-to-dictation 8 multiple-digit numbers (range of digits for each number: 4–6 digits). The total number of errors over 8 items is used in the analysis. Note that this measure was not available for 40 participants.

*Arabic number reading test*. This test is modified from the sub-test of Arabic number reading of the *Developmental Dyscalculia Battery* [120]. A list of 16 numbers is written in Times New Roman 14 and arranged in one column, printed on a sheet of paper. The number length varies from 3 to 6 digits and there are 4 numbers for each length. The digit “0” is present in an internal position in half of the numbers, to test the ability of the child to correctly resolve the role of the position of this digit in numbers such as “20056” or “4080”. The child has to read the list aloud, without time constraints. The time to complete the task and the number of errors is scored. However, only the number of errors over 16 items was entered into the analyses.

*Number Order test (from the AC-MT 6–11 battery* [119]*)*. This is a test of symbolic comparison estimation. The child has to order 10 series of 4 numbers printed on a sheet of paper (5 series from the largest to the smallest; 5 series from the smallest to the largest). The total number of incorrect series is scored. The number of wrong series over 10 trials was entered into the analyses.

*Arithmetic Facts test (from the developmental dyscalculia battery* [120]*)*. The examiner asks the child to say the result of sixteen multiplications (for example 3 times 8, 9 times 5, etc.) as rapidly as possible. The number of correct responses out of 16 trials is scored. Hesitations (silent pauses longer than 2 seconds) or attempts based on the use of a times table are scored and subtracted from the correct responses.

*Computation Strategies test (from the AC-MT 11–14 battery* [121]*)*. Written calculations are printed on a sheet of paper, and the result of each calculation is shown along with the calculation. Besides each complete calculation there is a similar calculation to be computed; this latter calculation may differ from the adjacent one by inversion of the terms, increase of one of the terms by addition of a unit (or multiplication by tens), substitution of one of the terms with the result, and so on. Thus, the child can determine the result of these operations without actually calculating them, but reasoning on the base of the similar complete calculations shown beside. The child is requested to perform rapidly (with an overall time constraint of 2 minutes) as well as accurately over a total of 16 trials. The number of computations performed correctly within the time limit was used in the analyses.

*Computation procedures (tabulation and carry)*. This novel task was made for the purpose of the present study. The text is based on 14 pre-filled written calculations in columns (3 additions, 3 subtractions, and 8 multiplications) printed on a sheet of paper. Each calculation is made by numbers in columns and includes the intermediate steps towards the solution (the result is not shown). The child has to say if the tabulation is arranged in a correct way or not. For example, the terms in an addition or the intermediate terms within a multiplication may not be correctly arranged in columns (i.e., one of the terms may be shifted toward the left, resulting in an incorrect tabulation of units, tens, and hundreds). In half of the trials, the operation is correct and in the other half, it is not. The child is requested to perform rapidly as well as accurately. The number of errors out of 14 trials was used in the analyses.

*Backward counting (from the AC-MT 6–11 battery* [119]*)*. The test assesses knowledge of the number line. To this aim, a task of backward counting is used. The child has to count out aloud backwards from 100 to 50 as rapidly and accurately as possible. One error score is considered every time the sequence is interrupted, regardless of the quantity of skipped consecutive numbers. The number of errors (out of 49 steps) was used for the analyses.

#### General cognitive predictors measures

Performance on the following general cognitive tests was considered in control models. The putative target dimension is presented in brackets:

*Symbol search subtest (from the WISC-R [122]; cognitive speed).* Sequences of 5 meaningless graphical symbols are arranged in 45 rows printed on a sheet of paper. For each string, the child has to respond by checking a YES or NO response box if that string contains one or both of the symbols presented as a research key to the left the string. The task must be completed within 2 minutes and the child is requested to work as rapidly as possible. The score is represented by the number of correct minus wrong responses within 2 minutes (skipped items or items that are not responded to because of the time limit are not included among wrong responses).

*Raven’s coloured progressive matrices (non-verbal intelligence)*. The number of correct responses is scored and used for the analyses.

*Forward and backward span of numbers (from the BVN battery [123]; verbal short-term memory).* The forward task requires the immediate serial recall of a sequence of digits. The examiner pronounces a sequence (one digit per second) and the child has to repeat the sequence in the same order. There are three sequences for each length, starting from the shortest (2 digits) to the longest (9 digits). The test ends when three sequences of the same length are not correctly recalled. The score (span) corresponds to the last length (i.e., the number of digits) for which at least two sequences were correctly recalled. The backward task and scoring are the same as the forward span, but the child has to recall each sequence in backward order. Both the forward and backward spans were entered in the analyses.

*Verbal phonemic fluency test (from the BVN battery, [123]; verbal fluency).* The child has to generate as many words as possible from the initial letters C, S, and P under the time constraint of 60 seconds for each letter condition. A short practice with a different letter (“T”) precedes the execution of the test. The experimenter asks the child to begin with the letter “C” and explicitly pronounces both the phonemes corresponding to that letter, that is /$\overset{⌢}{t\int }$/ (as in “cestino”, /tʃesˈtino/, “basket”) and /k/ (as in “cane”, /ˈkane/, “dog”). The number of correct items (excluding repetitions or variations of an already pronounced word) is scored.

### Procedure

Children were tested in a quiet room in their schools. Most tests were performed individually, while a few (Written Arithmetic Calculations, Number Order, and Raven’s Coloured Progressive Matrices) were administered collectively. Order of tests was fixed and was the following: MT reading test, RAN (colours and digits), "Nonna Concetta" Spelling-to-dictation, Verbal Phonemic Fluency, Mental Calculation, Backward Counting, Dictation of Numbers, Forward and Backward Span of Numbers, Raven’s Coloured Progressive Matrices, Number Order, Arithmetic Calculations (written), Symbol Search, Repetition of Pseudo-word Series, Arabic Number Reading, Orthographic Decision, Arithmetic Facts, Single Pseudo-word Repetition and Phonemic Segmentation, Computation Strategies, Computation Procedures, Orthographic decoding. (Visual-visual, Visual-auditory and Auditory-auditory Pseudo-word Matching tests). About three hours of testing were necessary to complete the battery. Most children completed testing in 3 sessions.

### Data analysis

To test the influence of predictors on reading, spelling and numerical skills we used the commonality analysis, a method of variance partitioning designed to identify proportions of variance in the dependent variable that can be attributed uniquely to each of the independent variables, and proportions of variance that are attributed to various combinations of independent variables ([124,125]. Notably, some of these interactions might also reveal suppressive effects, i.e., cases in which the predictor shares variance with another predictor and this variance does not contribute directly to performance on the dependent measure; the suppressive effect acts indirectly as a control of individual differences (such as differences in response speed) and typically increases the predictive power of the model. Communality analysis is thus a powerful analysis that is most effective in the case of a limited set of predictors. This particular feature is very useful here as our aim is to build models of performance characterised by both effectiveness (in terms of total variance explained) and parsimony (in terms of number of predictors used). Therefore, our approach was to evaluate the hypothesized cognitive dimensions using different combinations of the various markers adopted for each dimension to identify the most effective model and markers. In short, our approach was driven by hypotheses but was also flexible to detect the weight of different predictors.

Our general mode of proceeding in the analysis of data was as it follows. For each of the dependent measures (as defined above), we first carried out an analysis based on our hypotheses (as sketched in Fig 1). Notably, for some hypothesized dimension, we considered more than one possible marker (for the specific measures used, see the Results section). In this case, separate analyses were carried out for each of these markers and the best fitting model was considered. From this selection, we also obtained a list of tasks that provide the best marker for each behaviour. Then, we tested if the introduction of additional predictors appreciably increased the efficacy of the model. Final models are presented with tables and figures in the text while intermediate models are detailed in the Supporting Information. The results are also graphically illustrated by means of forest plots. This modality of presentation allows plotting the strength of the different betas (β), including the negative-suppressive ones, and provides at glance information about the capacity of the model to provide an overall explanation of the dependent measure.

We also tested the possible predictive power of general cognitive dimensions in two ways. On the one hand, we used the general cognitive dimensions to predict the target behaviour i.e., as predictors in a model of each of the dependent variable. The efficacy of these models was compared to that of the best model obtained by means of specific predictors (tables of models are provided also in this case). This allowed establishing whether the model based on specific markers of the hypothesized dimensions provided a better account of the target behaviour.

On the other hand, we separately added every single general cognitive dimension to the models (of reading, spelling and calculation) developed on the basis of specific predictors to see whether these variables allowed increasing the general power of the original model.

For each analysis, unique and common contributions are presented (either in a table or in a figure) along with other parameters, including the total variance explained by the model according to communality analysis, and the standardized β coefficients (and their significance values) of each predictor according to a regression analysis. Typically, the overall percentage of *R*^*2*^ explained by each variable is also presented providing an estimate of its overall influence. This refers to both the unique contribution of the variable and the contribution given in conjunction with other variables (referred as to common contribution). Note that, as the shared variance is considered for each variable, the sum of these values does not add to 100%.

## Results

### Descriptive analyses

Means, SDs, ranges and coefficients of variation for all variables are reported in S2 Appendix. Inspection of the table indicates that all variables produced a sufficiently wide range of variability to be included in the analyses. Furthermore, in no case there was the presence of marked ceiling or floor performance.

Correlations among all variables are reported in S3 Appendix.

### Analyses of specific predictors and predictors based on general cognitive dimensions

#### Reading fluency

First, we tested various multiple regression models on the dependent measure “reading fluency”. Based on our cognitive specific predictors (see Fig 1), we used a measure of “orthographic decoding” (via Visual-visual Pseudo-word Matching), one of “lexical efficiency” (namely, “Orthographic Decision”) and one relative to the “integration of reading sub-components” (namely, “RAN digits”). As measures of orthographic decoding, we separately tested different markers: pseudo-word matching in the visual-visual, in the visual-auditory or the auditory-auditory modalities (see below).

The analysis based on these three selected cognitive predictors is presented in Table 1 that reports the results of the multiple regression analysis on the dependent measure “reading fluency”. Unique and common contributions are summarized along with other parameters, including the total variance explained by the model (48.7%) according to communality analysis and the overall standardized β coefficients (and their significance values) according to a regression analysis. For the sake of presentation, we refer to this model as “MODEL 1”.

**Table 1:** MODEL 1: Reading fluency.

Reading (fluency)	*β*	*t*	*p*	Unique	Common	Total	% *R*^*2*^
Orthographic Decision	0.36	5.39	< .0001	0.19	0.10	0.29	60
RAN	0.47	6.82	< .0001	0.12	0.05	0.17	34
Visual-auditory Pseudo-word Matching	0.20	2.87	< .01	0.03	0.16	0.20	40

Unique, common, and total contributions for predictors of reading fluency. For each predictor (Orthographic Decision, RAN, and Visual-auditory Pseudo-word Matching), the overall standardized β coefficient, its *t* statistics and the significance *p* value (according to regression analysis) are reported as well as the percentage of variance explained in the communality analysis (% *R*^*2*^ = Total / *R*^*2*^).

MODEL 1: *R*^*2*^ = 0.487; *R*^*2*^ adjusted = 0.48; *F*_(3,128)_ = 39.53, *p* < .0001.

**Fig 2:**
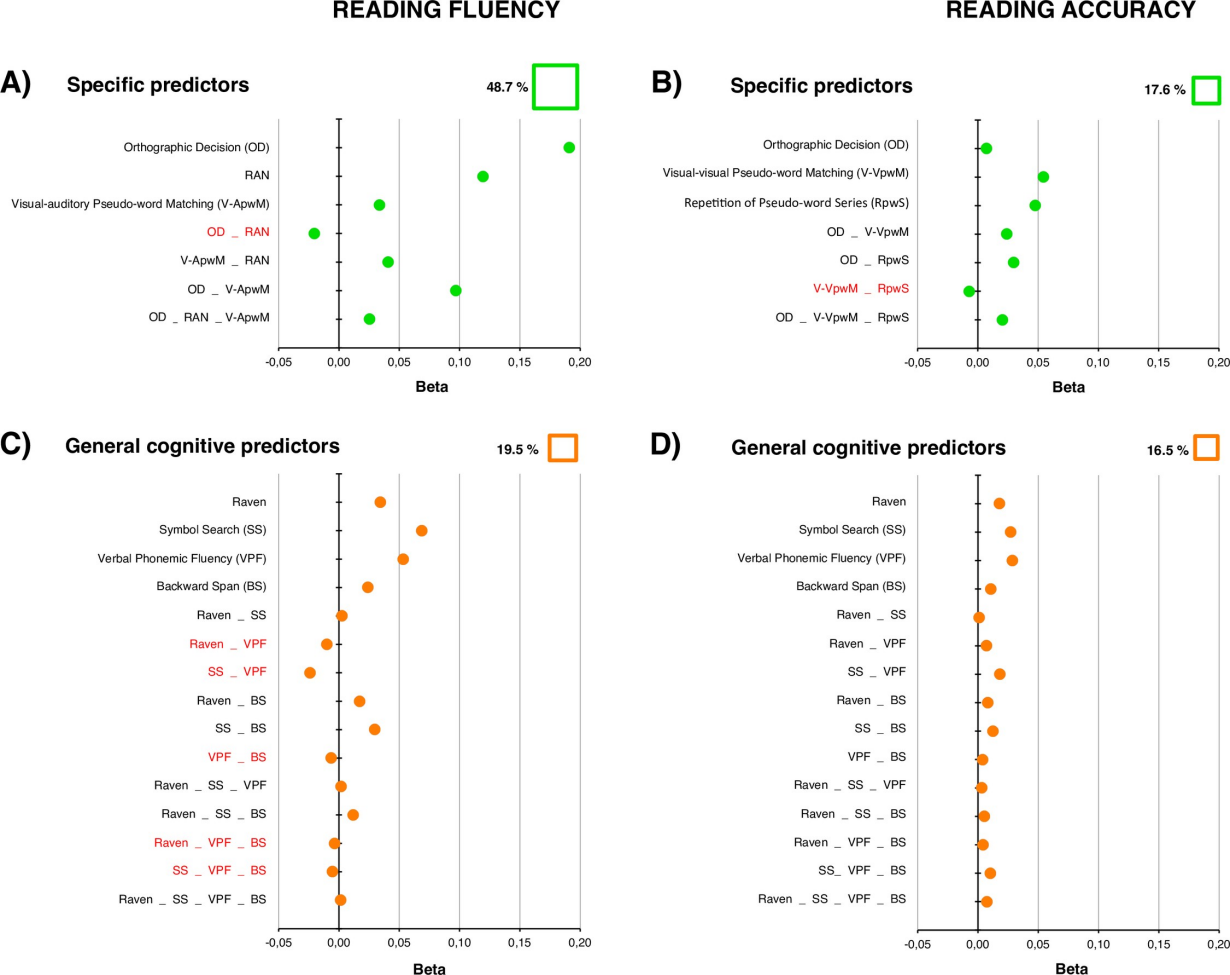
Forest plots relative to models of reading fluency (**A:** MODEL 1 using specific predictors) **and C** (MODEL 2 using general cognitive predictors) **and accuracy (B:** MODEL 3 using specific predictors) **and D** (MODEL 4 using general cognitive factors). Each plot shows the unique and common influences of the predictors on reading. The percentage of variance accounted by each model is indicated on the top right of each plot. The sizes of the green or orange squares beside the percentage of variance are proportional to the total amount of variance explained by each model. The green and orange dots (for the specific and general cognitive predictors, respectively) indicate the size of the beta coefficients for the unique or common portions of variance. Negative values (and red labels) mark suppressive effects.

The results of MODEL 1 are graphically illustrated in Fig 2A by means of a forest plot. Fig 2A shows the unique contributions of the three predictors as well as the common contribution of each pairs of factors and of all three factors together. Overall, the three factors account for more than 70% of the unique variance in reading fluency accounted by the model (which was 48.7% overall). The largest common contribution is that due to the communality between the Orthographic Decision and Visual-auditory Pseudo-word Matching tests (19.9%).

We run several other control analyses that are summarized in the text below and presented in details the Supporting Information. These analyses allowed us testing specific points, as indicated below.

One point regards the evaluation of the best test to predict reading fluency. We first run separate analyses using the three versions of the Pseudo-word Matching test. As compared to the analysis of MODEL 1, which used the Visual-auditory Pseudo-word Matching test and explained 48.7% of variance, the model including the Visual-visual Pseudo-word Matching test explained 45.7% and that including the Auditory-auditory Pseudo-word Matching explained 46.4%. In both cases, the unique contribution of the two tasks to the model was limited while the Visual-auditory Pseudo-word Matching test alone explained a higher percentage of variance (6.9%).

Another point regards the evaluation of the contribution of the phonological tests to predict reading fluency testing whether phonological tests added to the explained variance of MODEL 1. Thus, we run an analysis with the variables of MODEL 1 adding the Single Pseudo-word Repetition test (S1 Table). Results were similar when the Phonemic Segmentation test added to the variables of MODEL 1 (S1 Table).

A third point is the analysis of the effects of general cognitive dimensions. The possible predictive power of general cognitive dimensions was examined in two different ways. Firstly, we used these dimensions as predictors in a model of reading fluency (MODEL 2). Second, we separately added every single dimension to MODEL 1 to see whether these variables allowed increasing the general power of the model.

The model based on general cognitive factors (MODEL 2) is presented in Table 2 (part A) and illustrated in the forest plot in Fig 2C. Overall, the model accounts for 19.5% of variance in reading fluency. The difference in the predictive power of the models can be directly appreciated comparing plots A and C of Fig 2 (see also the relative size of the green and orange squares). The strongest unique influence is Symbol Search (β = .07), then Backward Span of Numbers (β = .05), then Raven (β = .03), and Verbal Phonemic Fluency (β = .02). However, Model 2 is also characterized by a complex pattern of interactions among predictors, including five different suppressive influences.

**Table 2:** Models of reading (fluency and accuracy), spelling (accuracy), and calculation (speed and accuracy) based on general cognitive factors.

	A. Reading (fluency)					B. Reading (accuracy)					C. Spelling (accuracy)					D. Calculation (speed)					E. Calculation (accuracy)				
	MODEL 2 (*R*^*2*^ = .195)					MODEL 4 (*R*^*2*^ = .165)					MODEL 6 (*R*^*2*^ = .065)					MODEL 8 (*R*^*2*^ = .128)					MODEL 10 (*R*^*2*^ = .128)				
	Un.	Com.	Tot	*p*	% *R*^*2*^	Un.	Com.	Tot	*p*	% *R*^*2*^	Un.	Com.	Tot	*p*	% *R*^*2*^	Un.	Com.	Tot	*p*	% *R*^*2*^	Un.	Com.	Tot	*p*	% *R*^*2*^
Raven	.03	.02	.06		32	.02	.04	.05	*	27	.04	.01	.06	*	80	0	0	0		1	.09	.02	.11	°	87
Symbol Search	.07	.02	.09	*	51	.03	.06	.08	°	42	0	0	0		0	.04	.05	.09	*	69	.01	.01	.02		18
Backward Span	.05	-.05	.01		4	.03	.05	.08	°	41	0	0	0		2	.03	.04	.07	*	52	0	0	0		3
Verbal Phonemic Fluency	.02	.04	.07	*	40	.01	.05	.06	*	31	.01	.01	.02		24	.01	.03	.03		27	0	.02	.03		20

Unique (Un.), common (Com.) and total (Tot) contributions of general cognitive predictors of text reading fluency (MODEL 2), reading accuracy (MODEL 4), spelling accuracy (MODEL 6), calculation speed (MODEL 8) and accuracy (MODEL 10). The Raven, Symbol Search, Backward Span, and Verbal Phonemic Fluency tests were as general cognitive predictors. For each predictor, the significant *p* values of *β* (according to regression analysis) are marked, and the percentage of variance explained in the communality analysis (% *R*^*2*^ = Total / *R*^*2*^) is reported.

* *p* < .05;

° *p* < .01.

Then, we replicated MODEL 1, including in each analysis one of the four general cognitive predictors. The four analyses (for Raven, Symbol Search, Backward Span of Numbers, and Verbal Phonemic Fluency, respectively) are presented in S2 Table. Overall, in none of the four analyses was there an appreciable increase in the total explained variance. Furthermore, none of the four variables exerted a unique contribution (maximum β = .002) when added to the variables of MODEL 1 while they accounted for some shared variance with other variables.

### Reading speed: Summary of findings and comments

The results indicate that three dimensions: “orthographic decoding”, “lexical efficiency” and “integration of reading sub-components” allow for a very substantial prediction of reading fluency. These findings confirm and extend our previous results on typically developing children [40]. In that study, we only considered “orthographic decoding” and “integration of reading sub-components” obtaining an explanation of 37% of variance in reading fluency. Here, we obtained similar results even without using vocal reaction times to measure “orthographic decoding” but relying on the number of correct responses within a time limit in pseudo-word matching. Notably, the model including the pseudo-word matching in the visual-auditory modality produced a stronger prediction than that based on the visual-visual version (and also the auditory-auditory version which we introduced as a control). This pattern of findings appears consistent with the proposal by Blomert [109] that the key decoding process concerns binding between orthographic and phonological traces. However, we tested which condition of orthographic decoding was the best predictor empirically; thus, replication of this finding is certainly in order.

Furthermore, unlike our previous investigation [40], we also tested here the role of lexical efficiency by using an Orthographic decision test. Results indicated that lexical efficiency was actually the strongest unique predictor in the model. This finding is in keeping with the idea that lexical processing is an important factor even in regular orthographies, such as Italian [49,50,51,52], for which generally reliance on lexical procedure occurs later and is less pronounced than in the case of irregular orthographies such as English [126].

Also, results on the possible role of general cognitive dimensions were clear. If one makes a model (MODEL 2) using only these general cognitive predictors obtains a moderate explanation of variance (19.5%). This is keeping with the large literature that shows differences between children with high or low reading skills in short term memory [12] or in perceptual speed [127]. However, the overall predictive power of MODEL 2 is appreciably smaller than MODEL 1. Furthermore, none of these variables measuring general cognitive dimensions allowed increasing the prediction obtained in MODEL 1 even though they shared variance with some of the predictors of this model. In the framework developed in the introduction, they act as “distal” factors on the dependent measure influencing it indirectly through the interaction with proximal predictors. Indeed, it is not difficult to understand that factors such as short-term memory or perceptual speed may modulate performance on tasks with a time limit such as the Visual-auditory Pseudo-word Matching test or the RAN test. However, the present results do not support the idea that these distal factors are critical to directly predict reading fluency.

### Reading accuracy

In a first analysis, as a model to account for reading accuracy we used the same predictors entered in MODEL 1 to predict reading fluency. However, the resulting model (presented in S3 Table) only explained 8.8% of variance and, in particular, there was no detectable effect, whether unique or common, of RAN. Therefore, we tried to develop–based on data analyses–an autonomous model for reading accuracy. The best combination of predictors included the Visual-visual Pseudo-word Matching test (“orthographic decoding”), the Orthographic Decision test (“lexical efficiency”) and a measure of “phonological representations”, i.e., the Repetition of Pseudo-word Series. The resulting MODEL 3 explains a total of 17.6% variance (Table 3 and Fig 2B). As reported in S4 Table, adding as a further variable the Phonemic Segmentation test did not change the variance explained, while adding the Single Pseudo-word Repetition test slightly raised the explanation to 19.2% (models not shown for the sake of brevity). One may also note that the effect of “lexical efficiency” was really very small.

**Table 3:** MODEL 3: Reading accuracy.

Reading (accuracy)	*β*	*t*	*p*	Unique	Common	Total	% *R*^*2*^
Orthographic decision	0.10	1.05	0.297	0.01	0.07	0.08	45
Visual-visual Pseudo-word Matching	0.25	2.87	< .01	0.05	0.04	0.09	51
Repetition of Pseudo-word Series	-0.24	-2.68	< .01	0.05	0.04	0.09	50

Unique, common, and total contributions for predictors of text reading accuracy (MODEL 3): Orthographic Decision, Repetition of Pseudo-word Series, and Visual-visual Pseudo-word Matching tests. For each predictor, the overall standardized β coefficient, its *t* statistics, and the significance *p* value (according to regression analysis) are reported as well as the percentage of variance explained in the communality analysis (% *R*^*2*^ = Total / *R*^*2*^).

MODEL 3: *R*^*2*^ = 0.176; *R*^*2*^ adjusted = 0.16; *F*_(3,128)_ = 8.91, *p* < .0001.

Similar to what done in the case of reading fluency, we tested the possible predictive power of general cognitive dimensions using them as predictors in a model of reading accuracy. The resulting MODEL 4 is presented in Table 2 (part B) and Fig 2D. Note that the overall explanation of this model (16.5% of variance) is similar to that produced using specific predictors. When each cognitive predictor was separately added to MODEL 3, the explained variance increased only in the case of Symbol search and Phonemic fluency task, passing from 17.6% to 21% and 23%, respectively (see S2 Table).

### Reading accuracy: Summary of findings and comments

The results indicate that a combination of the dimensions of orthographic decoding, lexical efficiency and phonological representations produce a model with low-moderate explanatory power of text reading accuracy (19.2%). Critically, a similar explanatory power was obtained with the model using general predictors (16.5%).

It is difficult to interpret in a conclusive way this finding and different factors may contribute to this outcome. It is well known that fluency is a more sensitive measure than accuracy in regular orthographies [60], possibly also because of ceiling effects. Thus, one very likely explanation for these results is simply that the dependent measure itself is not very sensitive in detecting individual differences particularly in typically developing readers, as in the present study. Another possibility is that we did not include the parameters that may be more sensitive to predict accuracy; however, the variables considered include the most quoted elements to explain reading and reading deficit in literature. At any rate, the prediction that can be obtained in the case of reading accuracy appears unsatisfactory.

### Spelling

Based on cognitive predictors described above and schematized in Fig 1, we initially examined models based on a measure of “lexical efficiency” and one relative to “phonological representations”. As to this latter factor, we had three different possible markers: Single Pseudo-word Repetition, Phonemic Segmentation, and Repetition of pseudo-word series. These initial analyses indicated that a model based on the Orthographic Decision and the Repetition of Pseudo-word Series tests explained 23.5% of variance. Using the Single Pseudo-word Repetition or the Phonemic Segmentation test to mark phonological processing yielded somewhat smaller effects (i.e., 22.3% and 20.4%, respectively).

**Fig 3:**
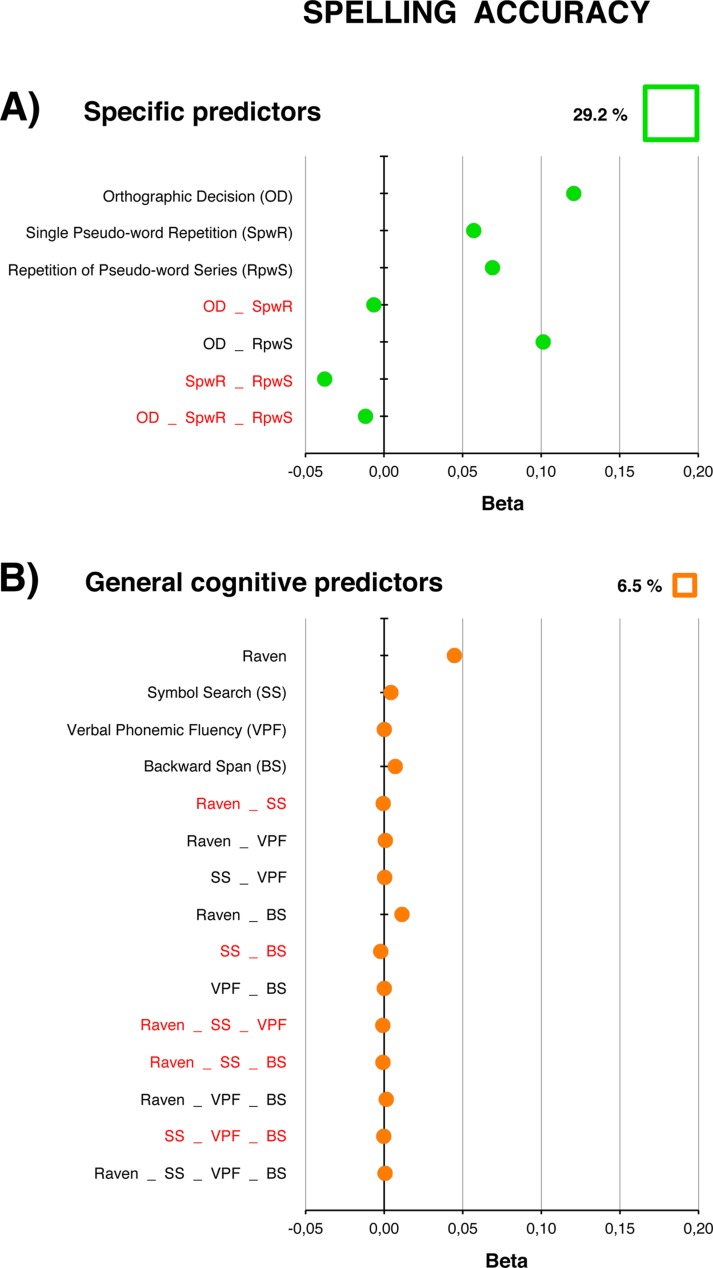
Forest plots relative to models of spelling accuracy. Each plot shows the unique and common influences of the predictors. The percentage of variance accounted by each model is indicated on the top right of each plot. Green or orange squares and dots, as well as negative values and red labels as in Fig 2. A) MODEL 5 showing the influences of the specific predictors. B) MODEL 6 using general cognitive factors as predictors.

Furthermore, we tried to examine whether a combination of phonological markers raised the overall power of the model. The best model (MODEL 5; Table 4 and Fig 3A) is based on the Orthographic Decision test (“lexical efficiency”) and on two different predictors from the “phonological representations” dimension, i.e., the Single Pseudo-word Repetition and the Repetition of Pseudo-word Series tests. Note that the influence of the Single Pseudo-word Repetition test is almost entirely suppressive, an effect exerted mostly on the Repetition of Pseudo-word Series test. Possible explanations of this finding are commented below. Overall, MODEL 5 explains 29.2% of variance in spelling.

**Table 4:** MODEL 5: Spelling.

Spelling	β	*t*	*p*	Unique	Common	Total	% *R*^*2*^
Orthographic Decision	0.38	4.62	< .0001	0.12	0.08	0.20	70
Single Pseudo-word Repetition	0.27	3.17	< .01	0.06	-0.06	0.00	0
Repetition of Pseudo-word Series	-0.32	-3.49	< .001	0.07	0.05	0.12	42

Unique, common, and total contributions for predictors of spelling (MODEL 5): Orthographic decision, Single pseudo-word repetition, and Repetition of Pseudo-word Series tests. For each predictor, the overall standardized β coefficient, its *t* statistics, and the significance *p* value (according to regression analysis) are reported as well as the percentage of variance explained in the communality analysis (% *R*^*2*^ = Total / *R*^*2*^).

MODEL 5: *R*^*2*^ = 0.292; *R*^*2*^ adjusted = 0.28; *F*_(3,128)_ = 17.19, *p* < .0001.

We tested whether adding measures of “orthographic decoding” produced any increase in the explanatory power of MODEL 5. The resulting models (S5 Table) indicate that performance on the visual-visual, visual-auditory, and auditory-auditory versions of the pseudo-word matching tasks did not contribute anything to the predictive power of MODEL 5. Similarly, no increase in power was obtained by adding the Phonemic Segmentation test.

Again, we tested the possible predictive power of general cognitive dimensions on spelling. Notably, the model using these measures (MODEL 6: Table 2 part C. and Fig 3B) has a very low explanatory power over spelling (total variance explained 6.5%). Consistently, adding the general cognitive factors did not produce any appreciable change to MODEL 5 (S2 Table).

### Spelling accuracy: Summary of findings and comments

The overall explanatory power of models for spelling is generally lower than that of reading fluency but a clear-cut distinction is observed between the model based on general cognitive factors (6.5%, MODEL 6) and the model based on the specific predictors (29.2%, MODEL 5) pointing to the effectiveness of a proximal model. According to this latter model, a combination of a measure of lexical efficiency and two measures of phonological processing provides a moderately good explanation of spelling performance.

As expected, no predictive role of the RAN task was detected on spelling. Furthermore, different measures of orthographic decoding did not contribute to variance in spelling performance. Finally, measures of phonological processing contributed to the prediction of spelling while they did not in the case of reading. In general, this latter finding is in keeping with the idea that spelling requires more fully specified phonological representations than does reading [42,43] and with the greater detrimental effect of acoustic-to-phonological difficulties on spelling with respect to reading [66]. Note that acoustic discriminatory skills did not contribute to explain spelling performance: when the auditory-auditory version of the Pseudo-word Matching test was added to the spelling model an increase of variance explained was not found.

In interpreting the phonological markers contributing to the prediction it should be considered that performance in the Repetition of Pseudo-word Series test predicted spelling directly while performance on the Single Pseudo-word Repetition test acted by suppressing variance in the Repetition of Pseudo-word Series test. Therefore, it appears that the quality of ongoing multiple representations (at work in the Repetition of Pseudo-word Series test) is critical to account for accurate performance in spelling upon dictation. In this vein, it should be kept in mind that in the dependent measure task (“Nonna Concetta”) the child listens to a short sequence of words (2–3 words) while attempting to spell the paragraph. Accordingly, one may envisage that dictation stresses the ability of the child to maintain in short term memory a complex sequence of phonological information. At the same time, it may also be noted that phonological working memory *per se* (as measured by the Span of Numbers test) does not seem able to carry on the critical relationship with spelling. Thus, performance on the Span of Numbers test (whether forward or backwards) did not exert an appreciable direct influence on spelling skills. Overall, results are in keeping with the idea that the critical phonological factor to predict spelling lies in an interaction between the quality of the phonological representations and the availability of this information in a short-term storage.

### Calculation skills

Separate analyses were carried out on a time measure of mental calculation and on an accuracy measure of both mental and written calculations.

First, we tested various multiple regression models on the dependent measure “calculation speed”. Based on our predictions, we used a measure of “number representation”, and one of “knowledge of arithmetic facts”. A first analysis, based on this prediction, generated a model with relatively good explanatory power (21.4%). However, as spelled out above, we also considered the potential role of a third variable, named “computation strategies” (represented by a dotted arrow in the sketch of Fig 1), and a number of other mathematical tasks, including knowledge of computation procedures, counting backwards, number reading. Therefore, we proceeded by adding each of these tasks to the model based on the basic prediction. These analyses indicated that introducing the factor “computation strategies” appreciably increased the explanatory power of the model. Therefore, in Table 5 (part A), we present the results of the multiple regression analysis on “calculation speed”, including this factor.

**Table 5:** MODEL 7 and MODEL 9: Calculation speed and accuracy.

	A: Calculation (speed)							B: Calculation (accuracy)						
	*β*	*t*	*p*	Un.	Com.	Tot.	% *R*^*2*^	*β*	*T*	*p*	Un.	Com.	Tot.	% *R*^*2*^
Number Order	0.01	0.17	0.87	0	0.08	0.08	21	0.18	2.14	< .05	0.03	0.11	0.14	50
Arithmetic Facts	-0.48	-6.13	< .0001	0.19	0.14	0.32	86	-0.23	-2.7	< .01	0.04	0.11	0.15	55
Computation Strategies	-0.24	-3.1	< .01	0.05	0.13	0.17	46	-0.28	-3.24	< .01	0.06	0.12	0.18	65

Unique, common, and total contributions for predictors of Calculation speed (MODEL 7) and accuracy (MODEL 9): Number Order, Arithmetic Facts, and Computation Strategies tests. For each predictor, the overall standardized β coefficient, its *t* statistics, and the significance *p* value (according to regression analysis) are reported as well as the percentage of variance explained in the communality analysis (% *R*^*2*^ = Total / *R*^*2*^).

A) Speed (MODEL 7): *R*^*2*^ = 0.379; *R*^*2*^ adjusted = 0.36; *F*_(3,128)_ = 25.38, *p* < .0001; B) Accuracy (MODEL 9): *R*^*2*^ = 0.275; *R*^*2*^ adjusted = 0.26; *F*_(3,128)_ = 15.81, *p* < .0001.

**Fig 4:**
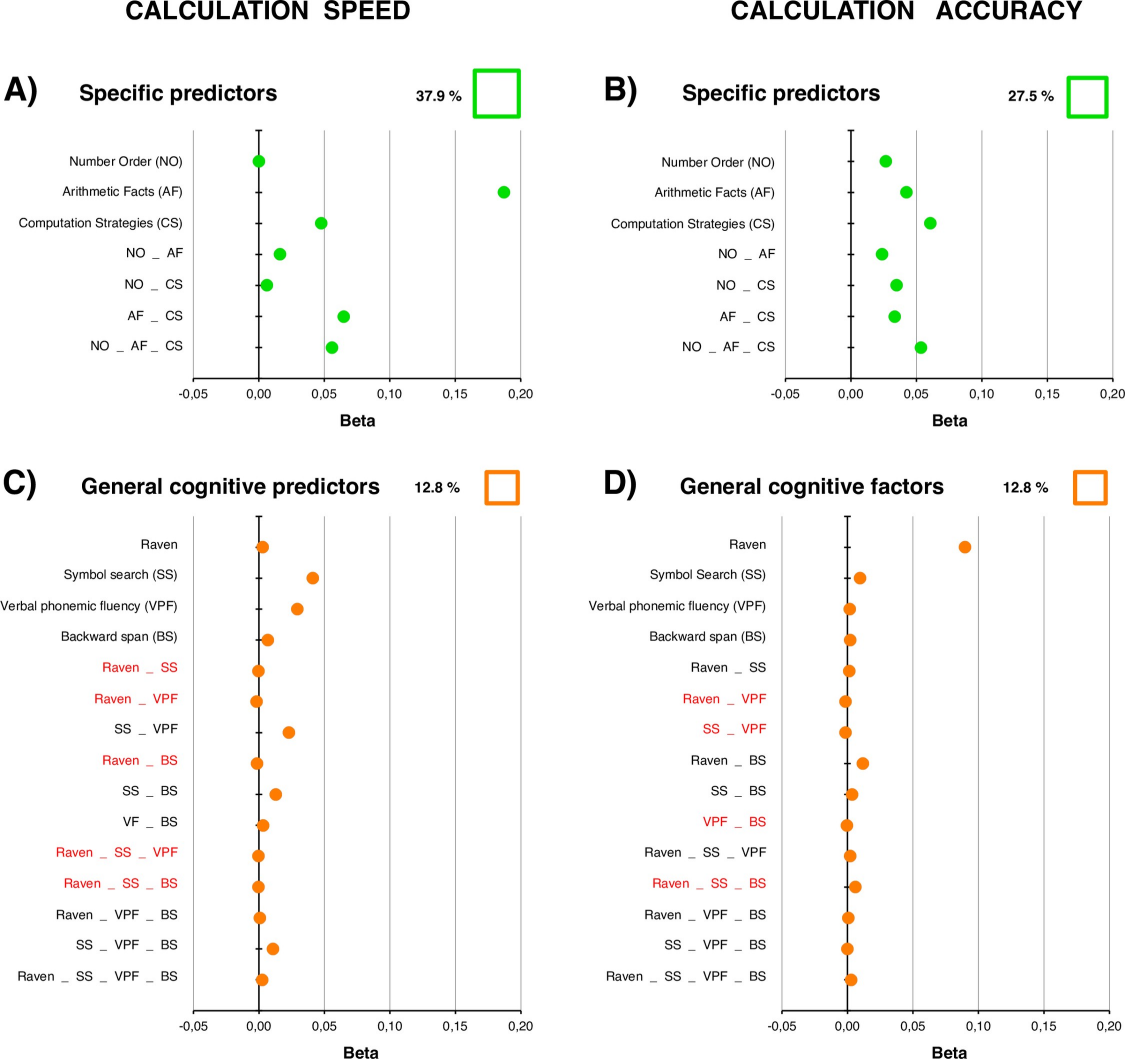
Forest plots relative to models of calculation speed **(A and C)** and accuracy **(B and D)**. Each plot shows the unique and common influences of the predictors on calculation. The percentage of variance accounted by each model is indicated on the top right of each plot. Green or orange squares and dots, as well as negative values and red labels as in Fig 2. A) MODEL 9 showing the unique and common influences of the specific predictors on calculation (speed). B) MODEL 10 using general cognitive factors as predictors of calculation (accuracy).

Table 5 (part A) reports the commonality coefficients and the percentage of variance for MODEL 7. Results are graphically presented in Fig 4A which shows the unique contributions of the three predictors as well as the common contribution of each pair of factors and of all three factors together. The model explains 37.9% of the total variance. It may be observed that the Computation Strategies test and, particularly, the Arithmetic Facts test exerted an influence on calculation speed both directly (particularly, knowledge of arithmetic facts which explains by itself 49.4% of variance) and in interaction with each other (17.1% of variance). The Number Order test does not exert a direct influence but only one in interaction with the other two factors.

Then, we replicated MODEL 7, including in each analysis one of the other control variables (i.e., the Computation Procedures test, the Backward Counting test, and the Arabic Number Reading and the Arabic Number Spelling test). None of the variables produced an increase in the variance explained by the model (in all cases the increase was less than 1%; see S6 Table—part A, for more details).

We then tested a model based only on general cognitive factors (MODEL 8). The model is presented in Table 2 (part D) and illustrated in Fig 4C. Overall, the model accounts for 12.8% of variance in calculation speed. There is some direct effect of the Symbol Search sub-test; however, the model is characterized by the presence of several, although very small suppressive interactions.

Then, we replicated MODEL 7, including in each analysis one of the four cognitive predictors (S2 Table). In three out of four analyses (Symbol Search sub-test, Verbal Phonemic Fluency test and Backward Span of Numbers test) the increase in the total explained variance was minimal (less than 1%). When the performance in the Raven test was added to the model, the power of the model increased passing from 37.9% to 40.7%. Notably, the performance on the Raven test exerted a suppressive influence on the Computation Strategies test and, secondarily on the Number Order test.

We then carried out the same analyses on the dependent measure “calculation accuracy”. Table 5 (part B) report the commonality coefficients and the percentage of variance for MODEL 9. Results are graphically presented in Fig 4B. The model is based on the same predictors as those of MODEL 7 and explains a total of 27.5% of variance. In this case, all three tests considered (“Number Order”, “Arithmetic Facts” and “Computation Strategies”) exerted some direct influence as well as some influence in interaction with each other.

Again, models made with the addition of control tests (Computation Procedures, Backward Counting, and Arabic Number Reading and the Dictation of Numbers) did not produce an appreciable increase in the explanatory power of the model (maximum increase = 1.1%) (see S6 Table—part B).

We then tested a model based only on general cognitive factors (MODEL 10). The model is presented in Table 2 (part E) and illustrated in Fig 4D. Overall, the model accounts for 12.8% of variance in calculation accuracy. By and large, the MODEL 10’s explanatory power is based on the unique influence of the performance in the Raven test. When we replicated MODEL 9, including in each analysis one of the four cognitive predictors (see S2 Table), an increase in the total explained variance was detected only for the Raven test (passing from 27.5% to 29.2%) and the Verbal Phonemic Fluency test (passing from 27.5% to 29.6%), due to the shared variance of Raven with all other predictors of MODEL 9, and to the suppressive effect of Verbal Phonemic Fluency on computation predictors.

### Math accuracy and speed: Summary of findings and comments

The model based on the factors hypothesized in the scheme of Fig 1 was moderately effective in predicting performance both in terms of accuracy (27.5%) and in the case of calculation speed (37.85%). Three main factors contributed to the prediction: “number representation”, “knowledge of arithmetic facts” and “use of strategy”. Note that the knowledge of computation procedures, as well as the ability of enumeration and of reading and spelling Arabic numbers did not contribute to the calculation efficiency, both in terms of accuracy and speed. Probably, at the age examined, these skills are already well acquired and automatized and do not contribute anymore to efficiency in calculation. Prediction based on the “specific” factors was considerably more effective than that based on general cognitive predictors both in the case of accuracy and in the case of speed. However, a small but clear suppressive effect was observed when the performance on the Raven test was added to the model of calculation speed based on the three specific factors.

## General discussion

With one exception (reading accuracy), the models developed to explain the abilities to read, spell and doing maths all explained a sizeable amount of variance; this ranged from 27.5% in the case of calculation (accuracy) to 48.7% of reading (fluency). Models based on general cognitive factors also accounted for some variance (ranging from 6.5% in the case of spelling to 19.5% in the case of reading fluency) but this was appreciably less than that explained by models based on the hypothesized proximal predictors. As stated above, the one exception was reading accuracy for which the models based on specific and general factors yielded similarly limited results. Furthermore, when added to the models of reading, spelling and maths, by and large, general cognitive predictors did not add substantial strength to the predictions. We can interpret these results as evidence that cognitive abilities measured by general predictors (such as memory, perceptual speed) act indirectly on the behaviours through a modulation of the efficiency of specific proximal predictors; in other terms, they can be seen as distal predictors.

**Fig 5:**
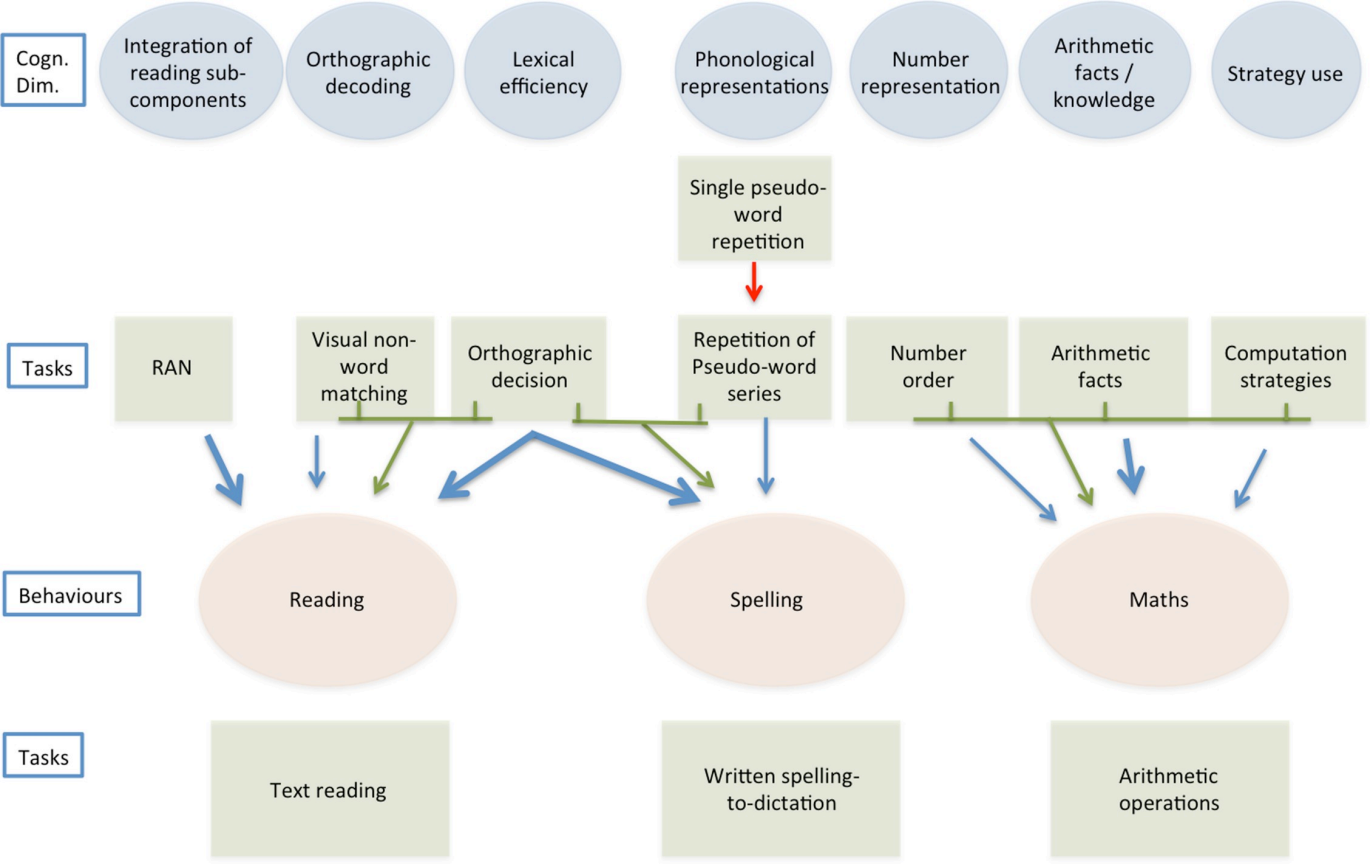
A proximal model of reading, spelling, and doing maths based on the main results of the present study. The figure presents the main links observed between tasks, used as predictors, and reading (fluency), spelling, and maths (accuracy) measures. Both direct links (in blue) and links expressing interactions (in green) between predictors are reported (for the sake of presentation only interactions with beta of ca. .05 or more are reported). The “heavier” blue arrows indicate “strong” influences (i.e., beta of ca. .10 or more). The red arrow indicates a suppressive effect.

Therefore, a first consideration is that the scheme presented in Fig 1 received some support from the results in the sense that it was able to predict sizeable proportions of variance in all but one dependent measure. Thus, models developed using a proximal approach i.e., making explicit the relation between each cognitive dimensions and the target behaviour, could effectively predict complex behaviours typical of everyday activities. These results are summarized in Fig 5 where the main results of the study are presented schematically.

Notably, this approach should be distinguished from the traditional approach of cognitive modelling. Indeed, these models do not focus on complex behaviours, such as reading a text, but rather aim to predict behaviours as expressed in quite “abstract” forms; for example, the dual route model is specifically aimed to predict reading aloud single monosyllabic words in English [7] but the authors did not explicitly state how to extend this proposal to the actual conditions which are used to test reading in a clinical setting (such as reading a meaningful text). In this vein, Bishop noted that “*traditional cognitive neuropsychology places a disproportionate emphasis on representational (competence) deficits*, *with processing (performance) deficits being relatively neglected*” [128]. This seems particularly relevant as it has been stressed that co-morbidity occurs among “complex behavioural disorders” [2]. In other terms, it seems likely that, in order to go beyond single behaviours and effectively explain comorbidity it is important to also model factors which contribute to actual processing (or performance factors in Chomsky’s perspective [129]).

As an example, we refer to RAN. In particular, we have proposed that this task captures the ability “*to integrate the sub-components involved in reading from multiple displays*”, an interpretation in line with an approach aimed to consider also processing factors [128]. Indeed, tasks such as RAN find no clear space in classical models of reading. However, if the aim is accounting for reading skills in a naturalistic context, such as reading a text or a story, RAN contributes to the prediction of reading fluency over and above measures of orthographic processing [28,40]. Notably, the results of the present study closely confirm these observations in that RAN contributed to the prediction of fluency in reading a text independently of visual-orthographic and lexical skills. Our proposal is that performance in RAN is specifically useful to comprehend the requirement, characteristic of functional reading that the child has to integrate and synchronize various sub-tasks, including visual scanning, word recognition, short-term memory, and pronunciation [24,130]. In Bishop’s perspective [128], skills in carrying out RAN tasks would account for “processing” factors characteristic of reading fluency in a naturalistic context. Seemingly, we have tried to incorporate other tasks into a general architecture of learning processes, hypothesizing links between each cognitive dimension and the target behaviours. Degree of specificity in the hypothetical links between behaviours, cognitive dimensions and tasks can be certainly refined but we propose that the scheme in Fig 1 and the resulting model summarized in Fig 5 may represent a useful, heuristic starting point to see representational and processing factors (in Bishop’s terms) in a unified proximal perspective.

Overall, we propose that an approach based on an analysis of proximal factors accounting for both representational and actual processing components of target behaviours is a promising venue to tackle learning disorders in general and, eventually, their co-morbidity. In particular, this perspective may favour linking more tightly the analysis of cognitive processes to the clinical measures used to test reading, spelling and maths.

It may be noted that this approach is different from traditional studies aimed to predict reading, spelling and maths (whether alone or in combination). We have proposed that many of these studies are envisaged within a distal perspective. By this, we mean that predictors are not framed within architectures of cognitive processes but rather causes are seen as separate from the processes accounting for performance in reading, spelling or doing maths. So, what it distinguishes the approach presented here is not so much the actual predictors used, which in several cases are common to previous studies, as much as the perspective in which these predictors are seen. In terms, we have tried to consider them in a proximal perspective, i.e., within a cognitive architecture of the target behaviour. As stated above, it must be stressed that predictors in themselves are neither proximal nor distal; what is proximal or distal is the perspective in which the relationship with the target behaviour is framed.

A focus of the present study was on accounting for the presence of associations (and hence co-morbidities) among different learning processes by identifying common predictors of the target behaviours in the perspective originally advanced by Pennington [2] with his multiple deficit model. The results provided some initial information in this respect. The Orthographic Decision test entered as a predictor of both reading and spelling, a finding consistent with the literature, which indicates that a single orthographic lexicon may account for performance in reading and spelling [48, 131, 132, 133]. However, apart from this, models for different skills were based on different factors. In particular, the factors selected to predict maths skills were entirely separate from those of reading and spelling. This selection is not surprising as, up until now, it has proven difficult to pinpoint the factors which account for the co-morbidity between reading and calculation disorders (e.g., [46]). One proposal that has been advanced is that phonological skills may account for such co-morbidity [45]. The present results did not offer much support to this proposal; in fact, phonological skills (in particular the Single Pseudo-word Repetition and Repetition of Pseudo-word Series tests) provided a relevant contribution only to predict spelling (MODEL 5). Therefore, it appears that more work is necessary to pinpoint the association between maths and literacy skills.

A number of limitations of the present study should be put forward. In the case of literacy measures, we focused on reading a text and spelling under dictation. There are certainly other measures which are relevant and are indeed included in clinical descriptions of learning disorders (e.g., in DSM-5) such as reading comprehension or writing a text (instead of spelling under dictation, as we tested). Presumably, including such dependent measures would require considering other additional predictors (such as morphological awareness and syntactic skills). Further research is needed to evaluate this possibility. Still, we propose that the general design used here may provide a useful base which may be expanded in the direction of examining additional dependent measures.

Another aspect of the present analyses that seems important to underscore is that we carried out a mixture of hypothesis-driven and data-driven analyses to establish the best models for each of the behaviours considered. More specifically, while we have proposed a general scheme of critical cognitive factors (dimensions in Fig 1) based on theoretical considerations, the actual best markers for each factor were established empirically choosing the predictors yielding the highest estimate. As an example of this, let us consider the predictors for phonological processing in the spelling model; both the Single Pseudo-word Repetition and Repetition of Pseudo-word Series entered in the model with the latter exerting a suppressive effect on the former. In interpreting this pattern, we focused on the characteristics of the dictation task, which was the target dependent measure. This features the dictation of a short series of words at a time. Thus, in keeping with Bishop’s distinction [128], one possible interpretation is that the Repetition of Pseudo-word Series captures variance closely associated to processing factors in a dictation task, while Single Pseudo-word Repetition may capture a level of ability of phonological processing, possibly independent of task characteristics. Thus, this interpretation rests on the idea that, among factors predictive of a learning behaviour, some may capture variance associated with representational components while other may point to the role of actual processing required by the specific task considered. Admittedly, this distinction between different phonological abilities is a post-hoc interpretation that certainly needs to be circumstantiated by further research. More generally, since evaluating various options for each model resulted in a large number of models being tested, the presents results should be certainly submitted to further testing and verification.

Finally, it should be underscored that the present study examined the performance of a group of typically developing children. As it is well known, performances on reading, spelling and calculation are described by continuous distributions and so-called “pathological” performances merely indicate performances lying at the very low ends of such continuous distributions (e.g., [108]). These considerations suggest the importance of studying also unselected groups of children, thus including many children in a typical range of performance; indeed, one may expect that associations and dissociations can be demonstrated both in the “normal” as well as in the “extreme” ranges of performance. Note that this approach is somewhat atypical. For example, models of reading, such as the DRC [7] or the CDP+ [8] have been especially developed in order to account for selective deficits in reading in both acquired and developmental disorders (and similar considerations apply to models of spelling and calculation). We propose that examining typically developing children may be a good starting point to construe a model of proximal factors which may account for the presence of both associations and dissociations among learning behaviours. Still, it is clearly important that in future research this model be tested on children with established mixed deficits in reading, spelling and calculation; this research project is currently underway.

All in all, these considerations mark the exploratory and heuristic nature of the present study which was guided by an interest in developing an initial sketch of a unitary architecture of reading, spelling and maths.

## Conclusions

Overall, based on the present results one might conclude that a) it was possible to develop models of reading, spelling and calculation using target behaviours functionally relevant in everyday life and using a proximal approach; b) these models were substantially “specific” because on the one hand they explained these behaviours with appreciably greater efficacy than general cognitive factors (distal factors, in the present terms) and, on the other hand, by using sets of predictors marking different putative dimensions for different behaviours; and c) the possibility of the model to explain comorbidity was limited to the association between reading and spelling while no specific information was detected on the overlap between reading/spelling and maths. However, further analyses of the present data may be carried out before this last conclusion is fully endorsed. The outcome of these further analyses will be the object of a future report.

## Supporting information

S1 Table Predictors of reading fluency: Original (MODEL 1) and alternatives. (DOCX)

S2 Table Original models (MODEL 1, 3, 5, 7 and 9) and addition of cognitive predictors. (DOCX)

S3 Table Reading accuracy predicted by predictors of reading fluency (MODEL 1). (DOCX)

S4 Table Predictors of reading accuracy: Original (MODEL 3) and alternatives. (DOCX)

S5 Table Another original model for reading accuracy (MODEL 5) and alternatives. (DOCX)

S6 Table Predictors of calculation: Original models (MODEL 7 and 9) and alternatives. (DOCX)

S1 Data (XLSX)

S1 Appendix [134,135]
(DOCX)

S2 Appendix Table with descriptive statistics. (DOCX)

S3 Appendix Intercorrelations table. (DOCX)
