# Supplementary material for: Predicting individual differences in reading, spelling and maths in a sample of typically developing children: A study in the perspective of comorbidity
Source: PLoS One. 2020 Apr 30;15(4):e0231937. doi: 10.1371/journal.pone.0231937 (PMC7192483; doi:10.1371/journal.pone.0231937)
Supplement: S1 Table — (DOCX) [file pone.0231937.s001.docx]

**S1 Table. Predictors of reading fluency: original (MODEL 1) and alternatives.**

| Reading (fluency) | *R^2^* total Model | *β* | *t* | *p* | Unique | Common | Total | % *R^2^* Tot. | % *R^2^* Un. | Shared variance with: |
| --- | --- | --- | --- | --- | --- | --- | --- | --- | --- | --- |
| ORIGINAL MODEL 1 | 0.487 |  |  |  |  |  |  |  |  |  |
| Orthographic Decision (OD) |  | 0.36 | 5.39 | < .0001 | 0.19 | 0.10 | 0.29 | 60 | 2 | -- |
| RAN |  | 0.47 | 6.82 | < .0001 | 0.12 | 0.05 | 0.17 | 34 | 39 | -- |
| Visual-auditory Pseudo-word Matching (V-ApwM) |  | 0.20 | 2.87 | < .01 | 0.03 | 0.16 | 0.20 | 40 | 7 | -- |
| MODEL 1 +  Single Pseudo-word Repetition (SpwR) | 0.488 | 0.04 | 0.55 | 0.58 | 0.00 | 0.08 | 0.08 | 16 | 0 |  |
| MODEL 1 +  Phonemic Segmentation (PS) | 0.497 | -0.12 | -1.57 | 0.12 | 0.01 | 0.10 | 0.11 | 23 | 2 | OD and  V-ApwM  (11%) |

Unique, common and total contributions for predictors of reading fluency in the original model (MODEL 1) and in the models obtained by adding phonological tests (Single Pseudo-word Repetition and Phonemic Segmentation tests, respectively). The column “Shared variance with” indicates the task(s) for which the shared variance of the model exceeded the 10% after a phonological test was added.
