# Supplementary material for: Predicting individual differences in reading, spelling and maths in a sample of typically developing children: A study in the perspective of comorbidity
Source: PLoS One. 2020 Apr 30;15(4):e0231937. doi: 10.1371/journal.pone.0231937 (PMC7192483; doi:10.1371/journal.pone.0231937)
Supplement: S2 Table — (DOCX) [file pone.0231937.s002.docx]

**S2 Table.** **Original models (MODEL 1, 3, 5, 7 and 9) and addition of cognitive predictors.**

Unique, common, and total contributions (both as raw coefficient of Unique, common, and total contributions (both as raw coefficient of explained variance and as % of *R^2^* respect to the total variance explained by a model) after that each cognitive predictor is added to an original model. Additionally, in the *p* column, the significance of β of each predictor according regression analysis is reported. Table reported also information about the percentage of explained variance by the original model and of the model after that each cognitive predictor have been added. In the column “Shared variance with” were reported task(s) for which the shared variance with the added predictor exceed the 10%.

Note: * *p* < .05; ° *p* <.01.

| Cognitive predictor added: | Original Model | | *% R^2^* original Model | *% R^2^* with the added predictor | Un. | Com. | Tot. | % *R^2^* Tot | % *R^2^* Un. | *p* | Shared variance with: |
| --- | --- | --- | --- | --- | --- | --- | --- | --- | --- | --- | --- |
| Raven | M. 1: Reading (fluency) | | 48.7 | 49 | 0.00 | 0.05 | 0.05 | 11 | 0 |  |  |
|  | M. 3: Reading (accuracy) | | 17.6 | 18 | 0.00 | 0.05 | 0.06 | 31 | 1 |  |  |
|  | M. 5: Writing | | 29.2 | 29 | 0.00 | 0.05 | 0.06 | 19 | 1 |  | OD and RpwS (13%) |
|  | M. 7: Calculation (speed) | | 37.9 | 41 | 0.03 | -0.03 | 0.00 | 0 | 7 | * |  |
|  | M. 9: Calculation (accuracy) | | 27.5 | 29 | 0.02 | 0.10 | 0.11 | 39 | 6 |  |  |
| Symbol Search | M. 1: Reading (fluency) | | 48.7 | 49 | 0.00 | 0.08 | 0.08 | 17 | 0 |  |  |
|  | M. 3: Reading (accuracy) | | 17.6 | 21 | 0.03 | 0.05 | 0.09 | 41 | 16 | ° |  |
|  | M. 5: Writing | | 29.2 | 31 | 0.01 | -0.01 | 0.00 | 0 | 4 |  |  |
|  | M. 7: Calculation (speed) | | 37.9 | 39 | 0.01 | 0.08 | 0.09 | 23 | 2 |  |  |
|  | M. 9: Calculation (accuracy) | | 27.5 | 28 | 0.00 | 0.02 | 0.02 | 9 | 0 |  |  |
| Backward Span | M. 1: Reading (fluency) | | 48.7 | 49 | 0.00 | 0.08 | 0.08 | 17 | 0 |  |  |
|  | M. 3: Reading (accuracy) | | 17.6 | 18 | 0.01 | 0.06 | 0.07 | 37 | 5 |  |  |
|  | M. 5: Writing | | 29.2 | 30 | 0.01 | 0.00 | 0.00 | 1 | 2 |  |  |
|  | M. 7: Calculation (speed) | | 37.9 | 38 | 0.00 | 0.07 | 0.07 | 18 | 0 |  |  |
|  | M. 9: Calculation (accuracy) | | 27.5 | 30 | 0.02 | -0.02 | 0.00 | 1 | 7 |  |  |
| Phonemic Fluency | M. 1: Reading (fluency) | | 48.7 | 49 | 0.00 | 0.06 | 0.06 | 13 | 0 |  |  |
|  | M. 3: Reading (accuracy) | | 17.6 | 23 | 0.05 | -0.04 | 0.01 | 3 | 22 | * |  |
|  | M. 5: Writing | | 29.2 | 30 | 0.01 | 0.01 | 0.02 | 6 | 2 |  |  |
|  | M. 7: Calculation (speed) | | 37.9 | 38 | 0.00 | 0.03 | 0.03 | 9 | 0 |  |  |
|  | M. 9: Calculation (accuracy) | | 27.5 | 28 | 0.00 | 0.02 | 0.03 | 9 | 1 |  |  |
|  |  | | | | | | | | | | |
