# Supplementary material for: Predicting individual differences in reading, spelling and maths in a sample of typically developing children: A study in the perspective of comorbidity
Source: PLoS One. 2020 Apr 30;15(4):e0231937. doi: 10.1371/journal.pone.0231937 (PMC7192483; doi:10.1371/journal.pone.0231937)
Supplement: S3 Table — (DOCX) [file pone.0231937.s003.docx]

**S3 Table.** **Reading accuracy predicted by predictors of reading fluency (MODEL 1).**

| A. Reading (accuracy) | Coefficient | Percent |
| --- | --- | --- |
| Unique to Orthographic Decision (OD) | 0.057 | 64.52 |
| Unique to RAN | 0.000 | .07 |
| Unique to Visual-auditory Pseudo-word Matching (V-ApwM) | 0.007 | 7.37 |
| Common to OD and RAN | 0.001 | .63 |
| Common to V-ApwM and RAN | 0.000 | .09 |
| Common to OD and V-ApwM | 0.025 | 27.84 |
| Common to OD and RAN and V-ApwM | -0.001 | -.53 |
| Total | 0.088 |  |

| B. Reading (accuracy) | *β* | *t* | *p* | Unique | Common | Total | *% R^2^* | |
| --- | --- | --- | --- | --- | --- | --- | --- | --- |
| Orthographic Decision | .26 | 2.79 | .006 | .06 | .02 | .08 | 93 |  |
| RAN | -.01 | -.09 | .930 | .00 | .00 | .00 | 0 |  |
| Visual-auditory Pseudo-word Matching | .09 | .94 | .347 | .01 | .02 | .03 | 35 |  |
|  |  |  |  |  |  |  |  |  |

(A) Commonality coefficients and percentage of explained variance for the predictors of reading fluency (MODEL 1) used to predict reading accuracy. (B) Unique and common contributions of predictors in the model for Reading accuracy.

R^2^ = .088; R^2^ adjusted = .07; F _(3,128)_ = 4.03, p < .01
