# Supplementary material for: Predicting individual differences in reading, spelling and maths in a sample of typically developing children: A study in the perspective of comorbidity
Source: PLoS One. 2020 Apr 30;15(4):e0231937. doi: 10.1371/journal.pone.0231937 (PMC7192483; doi:10.1371/journal.pone.0231937)
Supplement: S4 Table — (DOCX) [file pone.0231937.s004.docx]

**S4 Table. Predictors of reading accuracy: original (MODEL 3) and alternatives.**

| Reading (accuracy) | *R^2^* total Model | *β* | *t* | *p* | Unique | Common | Total | % *R^2^* Tot. | % *R^2^* Un. | Shared variance with: |
| --- | --- | --- | --- | --- | --- | --- | --- | --- | --- | --- |
| ORIGINAL MODEL (3) | 0.176 |  |  |  |  |  |  |  |  |  |
| Orthographic Decision (OD) |  | 0.10 | 1.05 | 0.297 | 0.01 | 0.07 | 0.08 | 45 | 6 | -- |
| Visual-visual Pseudo-word Matching (V-VpwM) |  | 0.25 | 2.87 | < .01 | 0.05 | 0.04 | 0.09 | 51 | 28 | -- |
| Repetition of Pseudo-word Series (RpwS) |  | -0.24 | -2.68 | < .01 | 0.05 | 0.04 | 0.09 | 50 | 28 | -- |
| MODEL 3 +  Single Pseudo-word Repetition (SpwR) | 0.192 | 0.15 | 10.59 | 0.12 | 0.02 | -0.02 | 0.00 | 1 | 9 |  |
| MODEL 3 +  Phonemic Segmentation (PS) | 0.18 | -0.05 | -0.47 | 0.64 | 0.03 | 0.07 | 0.09 | 51 | 14 | RpwS (13%); OD and  V-VpwM and RpwS (10%) |

Unique, common and total contributions for predictors of reading accuracy in the original Model (MODEL 3) and in the models obtained by adding phonological tests (Single Pseudo-word Repetition and Phonemic Segmentation tests, respectively). The column “Shared variance with” indicates the task(s) for which the shared variance with Pseudo-words repetition and Phonemic segmentation exceed the 10%.
