# Supplementary material for: Predicting individual differences in reading, spelling and maths in a sample of typically developing children: A study in the perspective of comorbidity
Source: PLoS One. 2020 Apr 30;15(4):e0231937. doi: 10.1371/journal.pone.0231937 (PMC7192483; doi:10.1371/journal.pone.0231937)
Supplement: S5 Table — (DOCX) [file pone.0231937.s005.docx]

**S5 Table.** **Another original model for Reading accuracy (MODEL 5) and alternatives.**

| Reading (accuracy) | *R^2^* total Model | *β* | *t* | *p* | Unique | Common | Total | % *R^2^* Tot. | % *R^2^* Un. | Shared variance with: |
| --- | --- | --- | --- | --- | --- | --- | --- | --- | --- | --- |
| ORIGINAL MODEL (5) | 0.292 |  |  |  |  |  |  |  |  |  |
| Orthographic Decision (OD) |  | 0.38 | 4.62 | < .0001 | 0.12 | 0.08 | 0.20 | 70 | 41 | -- |
| Single Pseudo-word Repetition (SpwR) |  | 0.27 | 3.17 | < .01 | 0.06 | -0.06 | 0.00 | 0 | 21 | -- |
| Repetition of Pseudo-word Series (RpwS) |  | -0.32 | -3.49 | < .001 | 0.07 | 0.05 | 0.12 | 42 | 24 | -- |
| MODEL 5 +  Visual-visual Pseudo-word Matching (V-VpwM) | 0.29 | 0.04 | 0.51 | 0.61 | 0.03 | 0.03 | 11 | 1 | 0 |  |
| MODEL 5 +  Visual-auditory Pseudo-word Matching (V-ApwM) | 0.30 | -0.07 | -0.79 | 0.43 | 0.00 | 0.01 | 2 | 1 | 0 |  |
| MODEL 5 +  Auditory-auditory Pseudo-word Matching (A-ApwM) | 0.29 | -0.01 | -0.11 | 0.91 | 0.01 | 0.01 | 2 | 0 | 0 |  |
| MODEL 5 +  Phonemic Segmentation (PS) | 0.29 | 0.04 | 0.41 | 0.68 | 0.00 | 0.01 | 0.01 | 4 | 0 |  |

Unique, common and total contributions for predictors of reading accuracy in the original Model (MODEL 5) and in the models obtained by adding the visual-visual, visual-auditory and auditory-auditory versions of the pseudo-word matching tasks, and the phonemic segmentation task to the original Model 6. The column “Shared variance with” indicates the task(s) for which the shared variance with the added predictors exceed the 10%.
