# Supplementary material for: Predicting individual differences in reading, spelling and maths in a sample of typically developing children: A study in the perspective of comorbidity
Source: PLoS One. 2020 Apr 30;15(4):e0231937. doi: 10.1371/journal.pone.0231937 (PMC7192483; doi:10.1371/journal.pone.0231937)
Supplement: S6 Table — (DOCX) [file pone.0231937.s006.docx]

**S6 Table. Predictors of calculation: original models (MODEL 7 and 9) and alternatives.**

|  | A: Calculation (speed) | | | | | | | | B: Calculation (accuracy) | | | | | | | |
| --- | --- | --- | --- | --- | --- | --- | --- | --- | --- | --- | --- | --- | --- | --- | --- | --- |
|  | *R^2^* total Model | Un. | Com. | Total | *% R^2^* Total | *% R2* Unique | *p* | Shared variance with: | *R^2^* total Model | Un. | Com. | Total | *% R^2^* Total | *% R2* Unique | *p* | Shared variance with: |
| ORIGINAL MODEL (7/9) | 0.379 |  |  |  |  |  |  |  | 0.275 |  |  |  |  |  |  |  |
| Number Order (NO) |  | 0 | 0.08 | 0.08 | 21 | 0 | ° | -- |  | 0.03 | 0.11 | 0.14 | 50 | 11 | ° | -- |
| Arithmetic Facts (AF) |  | 0.19 | 0.14 | 0.32 | 86 | 50 | * | -- |  | 0.04 | 0.11 | 0.15 | 55 | 14 | * | -- |
| Computation Strategies (CS) |  | 0.05 | 0.13 | 0.17 | 46 | 13 | * | -- |  | 0.06 | 0.12 | 0.18 | 65 | 21 | * | -- |
| MODEL 7/9 +  Computation Procedures (CP) | 0.38 | 0.00 | 0.03 | 0.03 | 9 | 0 |  | -- | 0.28 | 0.00 | 0.02 | 0.02 | 7 | 0 |  | -- |
| MODEL 7/9 + Backward Counting (BC) | 0.38 | 0.01 | 0.00 | 0.00 | 0 | 1 |  | -- | 0.28 | 0.00 | 0.01 | 0.01 | 4 | 0 |  | -- |
| MODEL 7/9 + Arabic Number Reading (ANR) | 0.38 | 0.01 | 0.08 | 0.09 | 23 | 1 |  | -- | 0.29 | 0.01 | 0.07 | 0.08 | 29 | 4 |  | -- |
| MODEL 7/9 + Arabic Number Spelling (ANS) | 0.38 | 0.00 | 0.00 | 0.00 | 1 | 0 |  | -- | 0.28 | 0.00 | 0.01 | 0.02 | 5 | 1 |  | -- |

Unique, common, and total contributions of predictors of calculation in the original Models (MODEL 7 and 9) and in the models obtained by adding the Computation Procedures, Backward Counting, Arabic Number Reading, and Arabic Number Spelling tests were added to the original models. The column “Shared variance with” ” indicates the task(s) for which the shared variance with the added predictors exceed the 10%.
