# Supplementary material for: Predicting individual differences in reading, spelling and maths in a sample of typically developing children: A study in the perspective of comorbidity
Source: PLoS One. 2020 Apr 30;15(4):e0231937. doi: 10.1371/journal.pone.0231937 (PMC7192483; doi:10.1371/journal.pone.0231937)
Supplement: S1 Appendix — [134,135] (DOCX) [file pone.0231937.s008.docx]

**Appendix A**

**Materials developed for the present research**

**Reading**

**Tests 4-5-6. Orthographic decoding: Visual-visual, Visual-auditory, and Auditory-auditory Pseudo-word Matching.**

**Test 4. Visual-visual pseudo-word matching.** This experimental test is comprised of 90 trials consisting of 90 pairs of pseudo-words presented in the visual modality (printed on a sheet of paper). In half of the pairs, both the pseudo-words are equal; in the other half, the pseudo-words of each pair differ by one letter. The length of the pseudo-words is 5, 6, and 7 letters (15 pseudo-word pairs per length). There are no items with double consonants and all bigrams in a pseudo-word are legal. Bigram frequency and the number of orthographic neighbors are counterbalanced among this test and those of Visual-auditory match and Auditory-auditory match. For the “different” pairs, each distracter is obtained by changing a vowel or a consonant. Note that the distracter is orthographically similar to the target (according to [134]) and that the two stimuli in the pair were comparable for bigram frequency and number of orthographic neighbors. In each equal or different pair, one item is written in capital letters and the other one in lower-case (e.g., ‘STADARA’, /staˈdara/ vs. ‘stapara’, /staˈpara/).

Pseudo-word pairs are written in Times New Roman 14 and arranged in two columns of 15 trials each, separately in three sheets of paper to form three experimental blocks of 30 trials each. On the right-hand side of each item, YES/NO response boxes are disposed. Pseudo-word length is counterbalanced across blocks. The order of blocks is fixed. In the first block, 80% of the trials are equal pairs; in the second, the proportion is 50%, and in the third, 20%. The order of equal and different pairs within each block follows a quasi-random order and is fixed.

The child has to read silently the pseudo-word pairs on the sheet of paper and to immediately indicate (by checking with a pen the YES or NO response box) whether they are or not the same. The child follows the trial order by columns on the sheet of paper. Before the beginning of the task, the child is told that reading aloud is not permitted. Four practice trials are administered to let the child familiarize with the test. Before the beginning of each block, the child is informed about the percentage of same and different pairs in that block. The child is requested to perform rapidly as well accurately, but there is no time constraint to complete the task. Both time and accuracy measures were collected. However, key analyses were based on the number of errors on the overall 90 trials.

**Test 5. Visual-auditory pseudo-word matching.** This experimental test is comprised of 90 trials consisting of 90 pairs of pseudo-words; for each pair, one item is presented in the auditory modality and the other one in the visual modality (that is, printed in a column on a sheet of paper). In half of the pairs, the auditory and visual items are equal; in the other half, they differ by one letter. The length of the pseudo-words is 5, 6, and 7 letters (15 pseudo-word pairs per length). There are no items with double consonants and all bigrams in a pseudo-word are legal. Bigram frequency and orthographic neighbors’ size are counterbalanced among this test and those of Auditory-auditory match and Visual-visual match. For the “different” pairs, each distracter is phonologically similar to the target by the change of a consonant with another one that differs for only one phonological feature (i.e., each couple of target-distractor is a minimal phonological pair; e.g., ‘dapra’, /ˈdapra/ vs. ‘dabra’, /ˈdabra/) maintaining the same CVCV structure. Target and distracter do not differ for bigram frequency and number of orthographic neighbors.

Pseudo-words in the auditory modality were read aloud by a speech therapist, whose voice was recorded and then processed offline using the Audacity 2.0.2 software to obtain three audio files (.aup format) suitable to be easily controlled by the experimenter using that same software during the test administration. In each trial, an acoustic warning (beep) alerts the child 500 ms before the auditory pseudo-word onset; a silent interval of 3 seconds follows the end of the pronounced item; then, a new warning is administered. In the visual modality, 90 items are written in Times New Roman 14 and arranged in two separated columns of 15 items each, similar to the visual-visual version of the test. The number of matched audio-visual pairs (correct response: YES) is 80% of the trials in the first block, 50% in the second, and 20% in the third. The order of equal and different pairs within each block follows a quasi-random sequence and was the same for every child (as was the sequence of blocks).

Before starting the three experimental blocks, four practice trials are administered to let the child familiarize with the test. Then, the experimenter starts the audio file to administer the sequence of trials to the child, who listen to the first warning and stimulus through headphones. After having listened to the first item, the child has to immediately indicate (by crossing with a pen the YES or NO response box) whether it was equal to the printed one. A 3-second silent interval provides the time to respond. Then, the second trial is presented and so on. The child must read the printed pseudo-words silently (the child is told before the beginning of the task that reading aloud is not permitted). The duration of the overall task is about the same for each subject (about 7 minutes), corresponding to the durations of the three audio files. Before the beginning of each block, the child is informed about the percentage of same and different pairs in that block. The child is also requested to perform rapidly and accurately. The accuracy measure used in the analyses is the percentage of errors on the overall 90 trials.

**Test 6. Auditory-auditory pseudo-word matching.** This test is comprised of 90 trials consisting of 90 pairs of pseudo-words presented in the auditory modality. In half of the pairs, the two pseudo-words are equal. All of the items are different from those of the Visual-auditory match test. The length of the pseudo-words is 5, 6, and 7 letters (15 pseudo-word pairs per length); there are no items with double consonants and all bigrams in a pseudo-word are legal. For the “different” pairs, distracters are made changing a phoneme with another one differing for only one phonological feature (as described for the visual-acoustic version). Targets and derived distracters did not differ from each other for bigram frequency and number of orthographic neighbors.

Pseudo-words were read aloud by four speech therapists, whose voice was recorded and processed using the Audacity 2.0.2 software to obtain three audio files (.aup format) corresponding to three experimental blocks. The structure of the presentation of each auditory stimulus in a trial is as follows: an acoustic warning (beep) alerts the child 500 ms before the onset of the first pseudo-word of the pair; this is followed by a second pseudo-word after a silent interval of 1 sec.; a silent interval of 3 seconds is set after the end of the second pseudo-word; then, a new warning is administered. The first pseudo-word of a pair is always pronounced by a different voice with respect to the second pseudo-word; the four voices are counterbalanced across blocks, pseudo-word length, and first or second item of a pair. Trials numbers are printed and arranged in two columns of 15 items each, in three sheets of paper to compose the response sheets corresponding to the three experimental blocks; on the right-hand side of each trial number, YES/NO response boxes are disposed. Pseudo-word length is counterbalanced across blocks. The order of blocks is fixed. The number of matched audio-audio pairs (correct response: YES) is 80% of the trials in the first block, 50% in the second, and 20% in the third. The order of equal and different pairs within each block is randomized and then fixed.

The experimenter starts the reproduction of an audio file to administer the sequence of trials to the child, who listens to a pair of stimuli at a time through headphones, follows the trial order by columns on the sheet of paper, and immediately indicates (by checking with a pen the YES or NO response box) whether the two items are the same or not. The time allowed to respond is provided by the 3-second silent interval following each pseudo-word pair in the audio file. The duration of the task is about 10 minutes, corresponding to the sum of the duration of the three audio files. Before starting with the three experimental blocks, four practice trials are administered to let the child familiarize with the test. Before the beginning of each block, the child is informed about the percentage of same and different pairs in that block. The child is also requested to perform rapidly as well as accurately. An accuracy measure based on the number of errors over the 90 trials was used for the analyses.

**Spelling**

**Tests 8-9. Single Pseudo-word Repetition and Phonemic Segmentation**. This is a new experimental test, created in our laboratory. A list of 30 long (from 8 to 10 letters) non-words (e.g., ‘zalvitodre’, /ˈdzalviˈtɔdre/) were presented in the auditory modality (a voice recorded on an audio file is reproduced by a computer software). The non-words were read aloud by a speech therapist, whose voice was recorded and processed using Audacity 2.0.2 software to obtain an audio file (.aup format) suitable to be easily controlled by the experimenter using that same software during test administration. Each stimulus was presented twice by the audio presentation in order to allow the repetition; afterwards, the child has to also perform the segmentation task on the same stimulus. Note that only the first repetition was considered for the scoring, while the second was administered only to ensure a correct listening of the stimulus (necessary for the segmentation task).

The structure of the presentation of each auditory stimulus in a trial is as follows: a non-word is delivered and an acoustic warning (beep) occurs 300 ms after the pronunciation offset; the experimenter stops the reproduction of the audio file upon the warning to allow the child executing the repetition of the item. Then, the experimenter releases the reproduction of the file to let the child listen to the non-word for the second time. Again, the acoustic warning beeps 300 ms after the non-word offset to allow the response of the child which now consists in a second repetition immediately followed by the segmentation (oral spelling) of the item, phoneme by phoneme (e.g., /dz/, /a/, /l/, /v/, /i/, /t/, /ɔ/, /d/, /r/, /e/); the child is not given a time limit to respond. The order of the stimuli is fixed. Before starting with the experimental list, three practice trials are administered to let the child familiarize with the test.

The experimenter notes the accuracy of the non-word repetitions and of the spelling; to avoid misunderstanding of the child’s response, the audio of his/her performance is also recorded allowing for offline checking. As reported above, the accuracy of non-word repetition is analyzed only for the first repetition. Accuracy of segmentation is measured as number of correct responses, both in terms of the number of correctly spelled non-words (N correctly spelled non-words/30: in a correctly segmented non-word, all the phonemes are spelled in the correct order, without omissions, intrusions, substitutions, or inversions of phonemes) and in terms of the total number of correctly spelled/sequenced phonemes (N correct phonemes/239, spelled in the correct order, regardless of possible intrusions). The number of correct first repetitions of a non-word (N correct repetitions/30), the number of correct segmentations and the number of correct segmented non-words were entered into the analyses.

**Test 10. Orthographic Decision**. This is a new test made for the present research: 40 words (mean length = 7.2 letters; SD = 1.9) with inconsistent spellings due to the presence of a phonemic segment with two homophonic transcriptions (only one of which is orthographically correct) were selected from a corpus of 1.000.000 stimuli taken from a word frequency dictionary for children [135].

Half of each experimental set was made of high-frequency words (mean = 232.8, SD = 441.8) and half of low-frequency words (mean = 5.8, SD = 4.9) according to Marconi et al.’s database [135]. As fillers, 15 regular words (i.e., words not containing any inconsistently spelled phonemic segment) were included; half were high-frequency words and half were low-frequency words.

Overall, the two sets of experimental words (high- and low-frequency inconsistently spelled words) were matched for length, bigram frequency (according to Marconi et al.’s database [135]), presence of consonant clusters and geminate consonants, presence of contextual rules, presence of morphologically complex words, percentage of non-dominant stress as well as for number of nouns, adjectives and verbs (all Fs < 1). Finally, the type of phonemic segment and the number of words with high vs low probability mapping was matched across sets.

A pseudo-word was created for each stimulus. Pseudo-words derived from inconsistently spelled words were pseudo-homophones (i.e., they resulted in a string that could be read as homophonous to the target; e.g., *QUOCO derived from CUOCO, cook - the asterisk marks nonlexical spelling errors). Thus, they can be detected only by relying on the lexical procedure. Pseudo-words derived from filler regular words resulted in strings that were non-homophonic because of the substitution or permutation of graphemes (e.g., ELEFANE derived from ELEFANTE, elephant). Thus, for these stimuli, lack of lexical value can be detected through both the lexical or sub-lexical procedures. The same types of error (insertions, deletions and substitutions) were introduced in the two sets of inconsistently spelled words (as well as in filler regular pseudo-words). There were no violations of Italian orthography; i.e., all pseudo-words were composed of legal letter sequences.

All stimuli (for a total of 110 stimuli, i.e., 15 filler words + 15 filler derived non-words; 40 words, and 40 derived pseudo-words) were presented printed on a sheet of paper in Times New Roman font (size 14) and arranged in two columns; on the right-hand side of each item, YES/NO response boxes are disposed. Correct and fake versions of each stimulus were never presented in the same block. The order of blocks is fixed.

The child has to read silently the words on the sheet of paper and to immediately indicate (by checking with a pen the YES or NO response box) whether or not they are correctly spelled. Before the beginning the task, the child is told that reading aloud is not permitted; six practice trials are administered to let the child familiarize with the test. The child is requested to perform rapidly as well accurately, but there is no time constraint to complete the task. The number of errors in judging the inconsistent items (both correct words and pseudo-homophones) is scored. The number of errors over 80 trials is used as a measure for the analyses.

**Test 11. Repetition of Pseudo-word Series** [118]. The test is comprised of 10 series of triplets of 3- and 5-letter pseudo-words presented in the auditory modality. The pseudo-words were read aloud by a speech therapist, and recorded and processed using the Audacity 2.0.2 software. The resulting audio file (.aup format) is used during the test administration using the same software. The structure of the presentation of each triplet of non-words in a trial is as follows: a non-word is delivered every 2 seconds; after the third one, an acoustic warning (beep) occurs (300 ms after the pronunciation offset); the experimenter stops the reproduction of the audio file upon the warning to allow the child repeating the triplet; then the experimenter releases the reproduction of the file to let the child listen to the following triplet, and so on.

The child is asked to listen to the triplet and repeat it in the same order immediately after the acoustic warning. The number of correctly repeated non-words (out of 30, independent of the sequence order) and the number of correctly repeated triplets (out of 10) is scored. The number of correct repetitions in 30 items was used in the analysis.
