# Supplementary material for: Predicting individual differences in reading, spelling and maths in a sample of typically developing children: A study in the perspective of comorbidity
Source: PLoS One. 2020 Apr 30;15(4):e0231937. doi: 10.1371/journal.pone.0231937 (PMC7192483; doi:10.1371/journal.pone.0231937)
Supplement: S2 Appendix — (DOCX) [file pone.0231937.s009.docx]

**Appendix B - Table with descriptive statistics**

| **Test** | **Unit of measure** | **N** | **Mean** | **SD** | **Coefficient of variation** | **Min** | **Max** | **Maximum possible score (closed scales only)** |
| --- | --- | --- | --- | --- | --- | --- | --- | --- |
| **Test 1. MT reading test (reading time)** | time per word (s) | 129 | 0.50 | 0.11 | 0.21 | 0.35 | 0.95 |  |
| **Test 1. MT reading test (accuracy)** | number of errors | 129 | 5.9 | 4.9 | 0.83 | 0.0 | 34.0 | n.a. |
| **Test 2. RAN colours** | time per item (s) | 129 | 0.78 | 0.16 | 0.21 | 0.46 | 1.30 |  |
| **Test 3. RAN digits** | time per item (s) | 129 | 0.50 | 0.12 | 0.24 | 0.32 | 1.00 |  |
| **Test 4. Orthographic decoding: Visual-visual Pseudo-word Matching** | number of errors | 129 | 2.4 | 3.7 | 1.59 | 0.0 | 27.0 | 90 |
| **Test 5. Orthographic decoding: Visual-auditory Pseudo-word Matching** | number of errors | 129 | 5.1 | 5.4 | 1.05 | 0.0 | 35.0 | 90 |
| **Test 6. Orthographic decoding: Auditory-auditory Pseudo-word Matching** | number of errors | 129 | 7.6 | 5.4 | 0.72 | 1.0 | 28.0 | 90 |
| **Test 7. "Nonna Concetta" Spelling-to-dictation** | number of errors | 129 | 2.8 | 2.2 | 0.81 | 0.0 | 12.0 | n.a. |
| **Test 8. Single Pseudo-word Repetition** | number of correct items | 129 | 16.0 | 4.4 | 0.27 | 4.0 | 25.0 | 30 |
| **Test 9. Single Pseudo-word Phonemic Segmentation** | number of correct segmentations | 129 | 187.9 | 23.0 | 0.12 | 116.0 | 223.0 | 239 |
| **Test 10. Orthographic Decision** | number of errors | 129 | 17.8 | 7.1 | 0.40 | 3.0 | 33.0 | 80 |
| **Test 11. Repetition of Pseudo-word Series** | number of correct items | 129 | 18.7 | 5.2 | 0.28 | 4.0 | 28.0 | 30 |
| **Test 13. Written Arithmetic Calculations** | time per item (s) | 129 | 41.8 | 12.9 | 0.31 | 17.5 | 90.9 |  |
| **Test 12-13. Mental and Written Arithmetic Calculations** | number of errors | 129 | 2.7 | 1.8 | 0.67 | 0.0 | 8.0 | 14 |
| **Test 14. Dictation of Numbers** | number of errors | 89 | 0.4 | 1.0 | 2.75 | 0.0 | 8.0 | 8 |
| **Test 15. Arabic Number Reading test** | number of errors | 129 | 1.6 | 1.7 | 1.04 | 0.0 | 8.0 | 16 |
| **Test 16. Number Order test** | number of errors | 129 | 1.6 | 1.3 | 0.82 | 0.0 | 7.0 | 10 |
| **Test 17. Arithmetic Facts test** | number of correct items | 129 | 13.2 | 3.0 | 0.23 | 1.0 | 16.0 | 16 |
| **Test 18. Computation Strategies test** | number of correct items | 129 | 12.6 | 3.3 | 0.26 | 4.0 | 16.0 | 16 |
| **Test 19. Computation Procedures (Tabulation and carry)** | number of errors | 129 | 2.1 | 2.3 | 1.07 | 0.0 | 9.0 | 14 |
| **Test 20. Backward Counting** | number of errors | 129 | 1.0 | 1.1 | 1.11 | 0.0 | 5.0 | 49 |
| **Test 21. Symbol Search subtest** | number of correct items | 129 | 27.4 | 4.1 | 0.15 | 16.0 | 39.0 | 45 |
| **Test 22. Raven’s Coloured Progressive Matrices** | number of correct items | 129 | 32.1 | 2.7 | 0.08 | 24.0 | 36.0 | 36 |
| **Test 23. Forward Span of Numbers** | span length (number of digits) | 129 | 5.0 | 0.9 | 0.17 | 4.0 | 7.0 | 9 |
| **Test 23. Backward Span of Numbers** | span length (number of digits) | 129 | 3.7 | 0.9 | 0.26 | 2.0 | 7.0 | 8 |
| **Test 24. Verbal Phonemic Fluency test** | number of correct items | 129 | 30.6 | 8.8 | 0.29 | 10.0 | 55.0 | n.a. |

n.a.: not applicable
