# Supplementary material for: Predicting individual differences in reading, spelling and maths in a sample of typically developing children: A study in the perspective of comorbidity
Source: PLoS One. 2020 Apr 30;15(4):e0231937. doi: 10.1371/journal.pone.0231937 (PMC7192483; doi:10.1371/journal.pone.0231937)
Supplement: S3 Appendix — (DOCX) [file pone.0231937.s010.docx]

**Appendix C - Intercorrelations table**

| **Pearson correlation matrix** | | **Test 1. MT reading test (reading time)** | **Test 1. MT reading test (accuracy)** | **Test 2. RAN colours** | **Test 3. RAN digits** | **Test 4. Orthographic decoding: Visual-visual Pseudo-word Matching** | **Test 5. Orthographic decoding: Visual-auditory Pseudo-word Matching** | **Test 6. Orthographic decoding: Auditory-auditory Pseudo-word Matching** | **Test 7. "Nonna Concetta" Spelling-to-dictation** | **Test 8. Single Pseudo-word Repetition** | **Test 9. Single Pseudo-word Phonemic Segmentation** | **Test 10. Orthographic Decision** | **Test 11. Repetition of Pseudo-word Series** | **Test 13. Written Arithmetic Calculations** | **Test 12-13. Mental and Written Arithmetic Calculations** | **Test 14. Dictation of Numbers** | **Test 15. Arabic Number Reading test** | **Test 16. Number Order test** | **Test 17. Arithmetic Facts test** | **Test 18. Computation Strategies test** | **Test 19. Computation Procedures (Tabulation and carry)** | **Test 20. Backward Counting** | **Test 21. Symbol Search subtest** | **Test 22. Raven’s Coloured Progressive Matrices** | **Test 23. Forward Span of Numbers** | **Test 23. Backward Span of Numbers** | **Test 24. Verbal Phonemic Fluency test** |
| --- | --- | --- | --- | --- | --- | --- | --- | --- | --- | --- | --- | --- | --- | --- | --- | --- | --- | --- | --- | --- | --- | --- | --- | --- | --- | --- | --- |
| **Test 1. MT reading test (reading time)** | | 1 | .301^**^ | .394^**^ | .406^**^ | .255^**^ | .443^**^ | .252^**^ | .299^**^ | -.283^**^ | -.336^**^ | .542^**^ | -.429^**^ | .534^**^ | .346^**^ | .000 | .308^**^ | .244^**^ | -.541^**^ | -.477^**^ | .130 | .181^*^ | -.291^**^ | -.232^**^ | -.254^**^ | -.249^**^ | -.287^**^ |
| **Test 1. MT reading test (accuracy)** | | .301^**^ | 1 | .080 | .015 | .303^**^ | .175^*^ | .082 | .287^**^ | -.036 | -.263^**^ | .285^**^ | -.301^**^ | .126 | .226^**^ | .055 | .212^*^ | .135 | -.185^*^ | -.223^*^ | .020 | .427^**^ | -.294^**^ | -.235^**^ | -.139 | -.262^**^ | .085 |
| **Test 2. RAN colours** | | .394^**^ | .080 | 1 | .709^**^ | .083 | .264^**^ | .080 | .063 | -.247^**^ | -.100 | .118 | -.268^**^ | .327^**^ | .175^*^ | -.059 | .159 | .223^*^ | -.423^**^ | -.175^*^ | .322^**^ | .056 | -.300^**^ | -.244^**^ | -.159 | -.118 | -.365^**^ |
| **Test 3. RAN digits** | | .406^**^ | .015 | .709^**^ | 1 | .031 | .225^*^ | .015 | -.037 | -.277^**^ | -.042 | .012 | -.134 | .391^**^ | .039 | -.128 | .000 | .030 | -.470^**^ | -.131 | .296^**^ | -.064 | -.312^**^ | -.006 | -.220^*^ | -.085 | -.353^**^ |
| **Test 4. Orthographic decoding: Visual-visual Pseudo-word Matching** | | .255^**^ | .303^**^ | .083 | .031 | 1 | .320^**^ | .373^**^ | .182^*^ | -.043 | -.245^**^ | .348^**^ | -.079 | .171 | .186^*^ | -.001 | .205^*^ | .125 | -.289^**^ | -.157 | .141 | .230^**^ | -.147 | -.264^**^ | -.075 | -.237^**^ | -.113 |
| **Test 5. Orthographic decoding: Visual-auditory Pseudo-word Matching** | | .443^**^ | .175^*^ | .264^**^ | .225^*^ | .320^**^ | 1 | .641^**^ | .081 | -.526^**^ | -.476^**^ | .347^**^ | -.438^**^ | .285^**^ | .278^**^ | -.029 | .218^*^ | .161 | -.463^**^ | -.175^*^ | .112 | .056 | -.241^**^ | -.286^**^ | -.170 | -.257^**^ | -.236^**^ |
| **Test 6. Orthographic decoding: Auditory-auditory Pseudo-word Matching** | | .252^**^ | .082 | .080 | .015 | .373^**^ | .641^**^ | 1 | .072 | -.429^**^ | -.361^**^ | .273^**^ | -.287^**^ | .241^**^ | .323^**^ | .000 | .152 | .157 | -.282^**^ | -.140 | .176^*^ | -.060 | -.141 | -.241^**^ | -.220^*^ | -.135 | -.153 |
| **Test 7. "Nonna Concetta" Spelling-to-dictation** | | .299^**^ | .287^**^ | .063 | -.037 | .182^*^ | .081 | .072 | 1 | .031 | -.115 | .451^**^ | -.347^**^ | .222^*^ | .340^**^ | .129 | .207^*^ | .367^**^ | -.318^**^ | -.282^**^ | .034 | .239^**^ | .010 | -.237^**^ | -.061 | -.130 | -.040 |
| **Test 8. Single Pseudo-word Repetition** | | -.283^**^ | -.036 | -.247^**^ | -.277^**^ | -.043 | -.526^**^ | -.429^**^ | .031 | 1 | .608^**^ | -.230^**^ | .479^**^ | -.206^*^ | -.127 | .051 | -.089 | -.198^*^ | .397^**^ | .080 | -.166 | .136 | .199^*^ | .286^**^ | .206^*^ | .118 | .318^**^ |
| **Test 9. Single Pseudo-word Phonemic Segmentation** | | -.336^**^ | -.263^**^ | -.100 | -.042 | -.245^**^ | -.476^**^ | -.361^**^ | -.115 | .608^**^ | 1 | -.296^**^ | .592^**^ | -.106 | -.247^**^ | -.090 | -.239^**^ | -.245^**^ | .336^**^ | .124 | .000 | -.079 | .093 | .375^**^ | .206^*^ | .288^**^ | .224^*^ |
| **Test 10. Orthographic Decision** | | .542^**^ | .285^**^ | .118 | .012 | .348^**^ | .347^**^ | .273^**^ | .451^**^ | -.230^**^ | -.296^**^ | 1 | -.413^**^ | .408^**^ | .417^**^ | .159 | .323^**^ | .495^**^ | -.518^**^ | -.428^**^ | .017 | .244^**^ | -.195^*^ | -.352^**^ | -.140 | -.293^**^ | -.273^**^ |
| **Test 11. Repetition of Pseudo-word Series** | | -.429^**^ | -.301^**^ | -.268^**^ | -.134 | -.079 | -.438^**^ | -.287^**^ | -.347^**^ | .479^**^ | .592^**^ | -.413^**^ | 1 | -.185^*^ | -.323^**^ | .002 | -.243^**^ | -.388^**^ | .380^**^ | .271^**^ | -.090 | -.146 | .255^**^ | .422^**^ | .249^**^ | .370^**^ | .293^**^ |
| **Test 13. Written Arithmetic Calculations** | | .534^**^ | .126 | .327^**^ | .391^**^ | .171 | .285^**^ | .241^**^ | .222^*^ | -.206^*^ | -.106 | .408^**^ | -.185^*^ | 1 | .389^**^ | .059 | .294^**^ | .281^**^ | -.570^**^ | -.418^**^ | .179^*^ | .028 | -.298^**^ | -.037 | -.197^*^ | -.186^*^ | -.259^**^ |
| **Test 12-13. Mental and Written Arithmetic Calculations** | | .346^**^ | .226^**^ | .175^*^ | .039 | .186^*^ | .278^**^ | .323^**^ | .340^**^ | -.127 | -.247^**^ | .417^**^ | -.323^**^ | .389^**^ | 1 | .124 | .290^**^ | .372^**^ | -.391^**^ | -.427^**^ | .142 | .100 | -.154 | -.336^**^ | -.147 | -.162 | -.062 |
| **Test 14. Dictation of Numbers** | | .066 | .024 | -.027 | -.114 | .008 | .107 | .134 | .119 | -.092 | -.168 | .247* | -.068 | .168 | .192 | 1 | .413** | .255* | -.258* | -.149 | .024 | .275** | .000 | -.239* | -.117 | -.059 | .075 |
| **Test 15. Arabic Number Reading test** | | .308^**^ | .212^*^ | .159 | .000 | .205^*^ | .218^*^ | .152 | .207^*^ | -.089 | -.239^**^ | .323^**^ | -.243^**^ | .294^**^ | .290^**^ | .365^**^ | 1 | .252^**^ | -.344^**^ | -.246^**^ | -.016 | .241^**^ | -.060 | -.299^**^ | -.043 | -.215^*^ | -.122 |
| **Test 16. Number Order test** | | .244^**^ | .135 | .223^*^ | .030 | .125 | .161 | .157 | .367^**^ | -.198^*^ | -.245^**^ | .495^**^ | -.388^**^ | .281^**^ | .372^**^ | .173^*^ | .252^**^ | 1 | -.361^**^ | -.388^**^ | .051 | .177^*^ | -.115 | -.448^**^ | .014 | -.111 | -.236^**^ |
| **Test 17. Arithmetic Facts test** | | -.541^**^ | -.185^*^ | -.423^**^ | -.470^**^ | -.289^**^ | -.463^**^ | -.282^**^ | -.318^**^ | .397^**^ | .336^**^ | -.518^**^ | .380^**^ | -.570^**^ | -.391^**^ | -.051 | -.344^**^ | -.361^**^ | 1 | .352^**^ | -.302^**^ | -.124 | .270^**^ | .196^*^ | .177^*^ | .224^*^ | .376^**^ |
| **Test 18. Computation Strategies test** | | -.477^**^ | -.223^*^ | -.175^*^ | -.131 | -.157 | -.175^*^ | -.140 | -.282^**^ | .080 | .124 | -.428^**^ | .271^**^ | -.418^**^ | -.427^**^ | -.074 | -.246^**^ | -.388^**^ | .352^**^ | 1 | -.116 | -.160 | .357^**^ | .351^**^ | .108 | .196^*^ | .239^**^ |
| **Test 19. Computation Procedures (Tabulation and carry)** | | .130 | .020 | .322^**^ | .296^**^ | .141 | .112 | .176^*^ | .034 | -.166 | .000 | .017 | -.090 | .179^*^ | .142 | -.042 | -.016 | .051 | -.302^**^ | -.116 | 1 | -.033 | -.141 | -.116 | -.064 | -.063 | -.135 |
| **Test 20. Backward Counting** | | .181^*^ | .427^**^ | .056 | -.064 | .230^**^ | .056 | -.060 | .239^**^ | .136 | -.079 | .244^**^ | -.146 | .028 | .100 | .290^**^ | .241^**^ | .177^*^ | -.124 | -.160 | -.033 | 1 | -.043 | -.225^*^ | -.101 | -.137 | .098 |
| **Test 21. Symbol Search subtest** | | -.291^**^ | -.294^**^ | -.300^**^ | -.312^**^ | -.147 | -.241^**^ | -.141 | .010 | .199^*^ | .093 | -.195^*^ | .255^**^ | -.298^**^ | -.154 | .038 | -.060 | -.115 | .270^**^ | .357^**^ | -.141 | -.043 | 1 | .143 | .115 | .321^**^ | .303^**^ |
| **Test 22. Raven’s Coloured Progressive Matrices** | | -.232^**^ | -.235^**^ | -.244^**^ | -.006 | -.264^**^ | -.286^**^ | -.241^**^ | -.237^**^ | .286^**^ | .375^**^ | -.352^**^ | .422^**^ | -.037 | -.336^**^ | -.164 | -.299^**^ | -.448^**^ | .196^*^ | .351^**^ | -.116 | -.225^*^ | .143 | 1 | .087 | .275^**^ | .196^*^ |
| **Test 23. Forward Span of Numbers** | | -.254^**^ | -.139 | -.159 | -.220^*^ | -.075 | -.170 | -.220^*^ | -.061 | .206^*^ | .206^*^ | -.140 | .249^**^ | -.197^*^ | -.147 | -.103 | -.043 | .014 | .177^*^ | .108 | -.064 | -.101 | .115 | .087 | 1 | .231^**^ | .084 |
| **Test 23. Backward Span of Numbers** | | -.249^**^ | -.262^**^ | -.118 | -.085 | -.237^**^ | -.257^**^ | -.135 | -.130 | .118 | .288^**^ | -.293^**^ | .370^**^ | -.186^*^ | -.162 | -.022 | -.215^*^ | -.111 | .224^*^ | .196^*^ | -.063 | -.137 | .321^**^ | .275^**^ | .231^**^ | 1 | .218^*^ |
| **Test 24. Verbal Phonemic Fluency test** | | -.287^**^ | .085 | -.365^**^ | -.353^**^ | -.113 | -.236^**^ | -.153 | -.040 | .318^**^ | .224^*^ | -.273^**^ | .293^**^ | -.259^**^ | -.062 | .137 | -.122 | -.236^**^ | .376^**^ | .239^**^ | -.135 | .098 | .303^**^ | .196^*^ | ,084 | ,218^*^ | 1 |
|  |  |  |  |  |  |  |  |  |  |  |  |  |  |  |  |  |  |  |  |  |  |  |  |  |  |  | |
|  |  |  |  |  |  |  |  |  |  |  |  |  |  |  |  |  |  |  |  |  |  |  |  |  |  |  | |
| ** p 0,01 (two-tailed). |  |  |  |  |  |  |  |  |  |  |  |  |  |  |  |  |  |  |  |  |  |  |  |  |  |  | |
| * p 0,05 (two-tailed). |  |  |  |  |  |  |  |  |  |  |  |  |  |  |  |  |  |  |  |  |  |  |  |  |  |  | |
